# Lung delivery of MSCs expressing anti-cancer protein TRAIL visualised with ^89^Zr-oxine PET-CT

**DOI:** 10.1186/s13287-020-01770-z

**Published:** 2020-06-26

**Authors:** P. Stephen Patrick, Krishna K. Kolluri, May Zaw Thin, Adam Edwards, Elizabeth K. Sage, Tom Sanderson, Benjamin D. Weil, John C. Dickson, Mark F. Lythgoe, Mark Lowdell, Sam M. Janes, Tammy L. Kalber

**Affiliations:** 1 Centre for Advanced Biomedical Imaging, Division of Medicine, University College London, London, UK; 2 Lungs for Living Research Centre, UCL Respiratory, Division of Medicine, University College London, London, UK; 3 Institute of Nuclear Medicine, University College London, London, UK; 4 Centre for Cell, Gene & Tissue Therapeutics, Royal Free Hospital, London, UK; 5 Department of Haematology, Cancer Institute, University College London, London, UK

**Keywords:** PET-CT, Cell tracking, TRAIL, ^89^Zr-oxine, Cord-derived MSCs, Cell therapy, Lung cancer

## Abstract

**Background:**

MSCTRAIL is a cell-based therapy consisting of human allogeneic umbilical cord-derived MSCs genetically modified to express the anti-cancer protein TRAIL. Though cell-based therapies are typically designed with a target tissue in mind, delivery is rarely assessed due to a lack of translatable non-invasive imaging approaches. In this preclinical study, we demonstrate ^89^Zr-oxine labelling and PET-CT imaging as a potential clinical solution for non-invasively tracking MSCTRAIL biodistribution. Future implementation of this technique should improve our understanding of MSCTRAIL during its evaluation as a therapy for metastatic lung adenocarcinoma.

**Methods:**

MSCTRAIL were radiolabelled with ^89^Zr-oxine and assayed for viability, phenotype, and therapeutic efficacy post-labelling. PET-CT imaging of ^89^Zr-oxine-labelled MSCTRAIL was performed in a mouse model of lung cancer following intravenous injection, and biodistribution was confirmed ex vivo*.*

**Results:**

MSCTRAIL retained the therapeutic efficacy and MSC phenotype in vitro at labelling amounts up to and above those required for clinical imaging. The effect of ^89^Zr-oxine labelling on cell proliferation rate was amount- and time-dependent. PET-CT imaging showed delivery of MSCTRAIL to the lungs in a mouse model of lung cancer up to 1 week post-injection, validated by in vivo bioluminescence imaging, autoradiography, and fluorescence imaging on tissue sections.

**Conclusions:**

^89^Zr-oxine labelling and PET-CT imaging present a potential method of evaluating the biodistribution of new cell therapies in patients, including MSCTRAIL. This offers to improve understanding of cell therapies, including mechanism of action, migration dynamics, and inter-patient variability.

## Introduction

Lung cancer is the leading cause of cancer death worldwide, survival rates are among the lowest [1], and improvement of treatment options is among the slowest of the major cancer types. The need for rapid translation and validation of new lung cancer therapies is therefore of high importance. Cell-based therapies have the potential to answer unmet clinical needs in a number of disease areas including oncology [2]. However, the spatial and temporal distribution of transplanted cells is rarely assessed in patients due to a lack of established technologies [3]. This can lead to concerns over safety and efficacy, lack of mechanistic understanding, and delays in translation [3–5]. We assess here the suitability of ^89^Zr-oxine labelling and positron emission tomography (PET) imaging to inform on the biodistribution of mesenchymal stromal cells (MSCs) in a clinical cell/gene-therapy trial for lung cancer.

Targeted stem cells expressing TRAIL as therapy for lung cancer (TACTICAL) is a prospective, randomised phase I/II trial to assess the safety and efficacy of third party, pooled human allogeneic umbilical cord-derived MSCs expressing TRAIL (MSCTRAIL) in combination with pembrolizumab, cisplatin, and pemetrexed as first-line therapy for metastatic lung adenocarcinoma [6, 7] (ClinicalTrials.gov Identifier: NCT03298763). TNF-related apoptosis-inducing ligand (TRAIL) selectively induces apoptosis in cancer cells via binding to cell surface death receptors (DR4, DR5), thereby activating the extrinsic apoptotic pathway [8]. TRAIL has also been shown to act synergistically with a range of chemotherapeutic drugs including cisplatin and pemetrexed, which act to upregulate death receptor expression, suiting TRAIL to use in combination therapy [9, 10]. Though the clinical utility of soluble recombinant TRAIL is limited by its short half-life in the body, its pharmacokinetics can be improved by constitutively expressing TRAIL on the surface of MSCs. This not only increases blood half-life but takes advantage of the reported tumour-tropism of MSCs [8, 11–13] and their propensity for lung delivery and retention following intravenous injection [14]. This study is a preclinical assessment of the feasibility of imaging lung-specific MSCTRAIL delivery, and duration of cell retention, using ^89^Zr-oxine and PET in a separate imaging arm of the TACTICAL phase II trial.

^89^Zr-oxine has recently emerged as a favourable PET alternative to ^111^In-oxine [15–17], which has enabled diagnostic tracking of white blood cell infusions for over 40 years with SPECT or scintigraphy. In addition to offering ^111^In-oxine’s advantages of ~ 3-day half-life and rapid radiolabelling, ^89^Zr-oxine benefits from the > 10-fold increased sensitivity of detection associated with PET [18]. With the introduction of total-body clinical PET scanners, this is set to increase by a further ~ 40-fold, reducing scan times and radioactive doses for patients and thus increasing the practicality of whole-body cell tracking in the clinic [19]. A handful of preclinical studies have so far shown the worth of ^89^Zr-oxine in tracking cell therapies in mouse models, including T cells [20, 21], dendritic cells, NK cells [17], and bone marrow cells [22, 23]. However, ^89^Zr-oxine toxicity is dependent on the amount used for labelling and varies between cell types, requiring individual evaluation with each prospective cell therapy prior to clinical implementation.

Therapeutic and phenotypic equivalence after ^89^Zr-oxine labelling was shown for MSCTRAIL, and toxicity investigated to ascertain tolerated labelling amounts in vitro. Delivery of viable cells and their retention in the lung was shown over 7 days in a preclinical lung tumour model, and human dosimetry estimates were calculated. This study illustrates the feedback that ^89^Zr-oxine labelling and PET imaging could provide on the biodistribution of cell-based therapies during the clinical trial phase of development. Wider use of cell tracking techniques such as this promises to contribute to a better scientific understanding of therapeutic cell behaviour within patients, as well as their associated safety profile, thereby informing future developments and effective translation.

## Methods

### ^89^Zr-oxine synthesis and purification

^89^Zr-oxalate stock (2–40 MBq; Perkin Elmer) was diluted to 500 μL with HPLC-grade water and neutralised with 1 M NaOH (Sigma-Aldrich). To this was added 20 μL of a 10-mg/mL solution of 8-hydroxyquinoline (ACS reagent, 99%, Sigma-Aldrich: 252565) in chloroform (Sigma-Aldrich), in a screw-top round-bottomed tube (BRAND® culture tubes, 6.5 mL, AR-Glas®, Sigma-Aldrich) and held onto a vortex mixer (Grant Bio, model PV-1) for 5 min using a clamp. Chloroform was added (480 μL) before vortexing for 25 min. After brief centrifugation, the chloroform phase containing Zr-oxine was removed and evaporated at 80 °C in a conical bottom HPLC vial (Supelco CD vial, 9-mm screw, Sigma-Aldrich), before resuspension in 15 μL of dimethyl sulfoxide (DMSO anhydrous, Sigma-Aldrich) at 50 °C for 20 min. Radiochemical yield (RCY) was calculated as the percentage of total original activity extracted into the chloroform phase, giving an average RCY of 74.2% ± 4.3 SEM (*n* = 16). Negative control synthesis was performed without addition of 8-hydroxyquinoline to the chloroform phase, resulting in retention of all the activity in the aqueous phase.

### MSC isolation

Human umbilical cord tissue was manually dissected, then enzymatically and mechanically digested to isolate MSCs using plastic adherence to cell culture-treated flasks. Passage 0 cultures were cultured in serum-containing α-MEM (Gibco) with antibiotic/antimycotic, then expanded subsequently in serum-free, xeno-free medium prior to labelling. A pooled population of MSC TRAIL derived from three donor cords was used for all experiments, except for those shown in figure S2, which were from a single donor.

### Cell culture

Cells were cultured in 5% CO_2_, at 37 °C, in either RPMI (Gibco), supplemented with 10% foetal bovine serum (FBS; Gibco) for PC9 (lung adenocarcinoma) CRL2081, H28, H2869 (malignant pleural mesothelioma), and MDAMB-231 (breast adenocarcinoma) cells, or α-MEM (Gibco) with 10% FBS (Gibco) for uct-MSCs (MSCTRAIL). α-MEM was also used for MSC/cancer cell co-culture apoptosis assays. H28, PC9, and H2869 lines were obtained from the Wellcome Trust Sanger Institute, and MDAMB-231, CRL-2081, and 293T were obtained from Cancer Research UK. All tissue culture reagents were obtained from Invitrogen. For cell culture following radiolabelling, growth media were supplemented with 50 U/mL penicillin and 50 mg/mL streptomycin (Invitrogen). MSCTRAIL and untransduced uct-MSCs were passaged once they reached 70 to 80% confluency. Cells were washed twice with PBS and incubated with trypsin (0.05% with EDTA, Gibco) at 37 °C until detached, and trypsin removed after dilution with media by centrifugation at 200*g*, before resuspension of cells in fresh media. MSCs were re-seeded at 500 to 3000/cm^2^ depending on the assay.

### Lentiviral production and transduction

The lentiviral plasmid expressing TRAIL, pCCL-CMV-flT, previously described [24] was used to overexpress TRAIL on MSCs. The ZsGreen-luciferase plasmid, pHIV-Luc-ZsGreen (a gift from Bryan Welm, Addgene plasmid #39196), was used for generating ZsGreen luciferase-expressing lentivirus. Lentiviral vectors were produced by co-transfection of 293T cells with construct plasmids together with the packaging plasmids pCMV-dR8.74 and pMD2.G using DNA transfection reagent jetPEI (Source Bioscience UK Ltd). Lentiviruses were concentrated by ultracentrifugation at 17,000 rpm (SW28 rotor, Optima LE80K Ultracentrifuge, Beckman Coulter, Brea, CA) for 2 h at 4 °C. Titres were determined via transduction of 293T cells with serial dilutions of virus and 8 μg/ml Polybrene (Sigma-Aldrich). TRAIL and ZsGreen expression were assessed by flow cytometry. MSCs were transduced at a range of MOIs using 8 μg/ml Polybrene and transduction efficacy assessed by flow cytometry.

### Cell labelling

MSCTRAIL (1.5 × 10^6^ per condition for toxicity experiments; 2 × 10^7^ for in vivo injections) were resuspended in 200 μL PBS. A further 100 μL was added containing PBS + ^89^Zr-oxine resuspended in DMSO (3% final concentration per 300 μL in both ^89^Zr-oxine and sham labelling conditions), with a further control of PBS alone. Cell suspensions were incubated for 20 min at room temperature, pelleted at 900 g, supernatant removed, and resuspended in PBS. This was repeated three times to remove unbound radiolabel. Final bound activity was measured using an ionisation chamber (Curiementor 4, PTW Freiburg GmbH) and expressed as kBq per 10^6^ cells, following cell counting. Total labelling and washing time was ~ 45 min on each occasion.

### Flow cytometry

For flow cytometry detection of TRAIL expression, cells were stained with a 1:50 dilution of phycoerythrin (PE)-conjugated mouse monoclonal antibody against human TRAIL (550516, BD Biosciences). For immunophenotyping of MSCs, the cells were stained with 1:50 dilution of fluorochrome-conjugated mouse monoclonal antibodies against CD73 (PE-Vio770, 130-104-224, Miltenyi Biotec), CD105 (APC, 130-094-926, Miltenyi Biotec), CD90 (PE, 561970, BD Biosciences), CD34 (FITC, 560942, BD Biosciences), CD45 (FITC, 560976, BD Biosciences), and HLA-DR (FITC, 555560, BD Biosciences).

### In vitro cell viability assays

MSCTRAIL were labelled as above with a range of ^89^Zr-oxine amounts dissolved in DMSO, or DMSO only or PBS only for the control conditions. Cells were seeded into 96-well culture plates at 10,000/well in 100-μL culture medium straight after radiolabelling, *n* = 4 per condition. A separate plate was seeded for each time point and assay. For ATP measurement, CellTiter-Glo® reagent was freshly prepared and added at a 1:1 ratio to culture medium. Light output was measured using a plate reader (VarioSkan Lux, Thermofisher Scientific) 10 min after reagent addition. For measurement of the reducing power of cells (NADH levels), XTT reagent (Roche) was prepared according to the manufacturer’s instructions. To each well, 50 μL reagent was added, including control wells containing media but no cells, followed by 4-h incubation at standard culture conditions. Absorbance at 490 nm and 630 nm was recorded using a plate reader (VarioSkan Lux, Thermofisher Scientific), and mean absorbance of each well at 630 was subtracted from absorbance at 490 nm and corrected for background metabolism in control wells. For measuring the cell viability of cancer cells upon co-culture with MSCTRAIL, ZSGreen luciferase-expressing H28 and PC9 tumour cells were seeded into 96-well culture plates at 5000 cells/well in 100 μL culture medium and treated with 2000 MSCTRAIL for 24 h. To measure cell viability, luciferin was added to each well (10 μL, 15 mg/mL) and photon counts were obtained after 10 min using a plate reader (VarioSkan Lux, Thermofisher), with 1-s acquisition per well. Each condition was obtained in triplicate.

### Activity retention assay

MSCTRAIL were seeded at 10,000/well in α-MEM (Gibco) with 10% FBS in 96-well plates. At each time point, growth media were removed and pooled with a wash of 100 μL PBS (+ 1 mM EDTA). Cells were lysed using 100 μL RIPA buffer (Thermo Scientific), which was pooled with a further wash of 100 μL PBS (+ 1 mM EDTA). Activity in media and cell fractions was measured using a Wizard 2480 automated gamma counter (PerkinElmer), and the percentage of activity retained calculated as the percentage of the total amount in the cell + media fractions in the cell fraction. Four individually seeded replicates were taken per time point.

### In vitro apoptosis assay

To assess the apoptosis of tumour cells co-cultured with MSCTRAIL, DiI-labelled cancer cells were plated into a 96-well plate (5000 cells/well), to which 2000 MSCTRAIL cells were added for 24 h. Floating and adherent cells were stained with AF647-conjugated Annexin V (Invitrogen) and 2 μg/mL DAPI (Sigma) and were assessed by means of flow cytometry. Annexin V+ cells were considered to have undergone apoptosis; Annexin V+/DAPI+ cells were considered to be dead by apoptosis.

### Western blot

Cells labelled as above were seeded at a density of 7.5 million into T175 flask (Nunc) and split 1 in 5 at 72 h. The remaining cells were pelleted and snap frozen using powdered dry ice. Re-seeded cells were harvested at 7 days post-labelling and frozen in the same way. Frozen samples were left to decay until no background radiation was detectable, and protein lysates prepared using cell lysis buffer (RIPA, Thermofisher Scientific) according to the manufacturer’s instructions. Protein content of cell lysates was measured using the BCA assay according to the manufacturer’s instructions (Pierce, Thermofisher Scientific). Lysates were denatured using 10x reduction buffer (NuPage Sample reducing agent, Life Technologies) and loaded onto gels (BioRad Mini Protean TGX) at 30 mg together with 4x loading buffer (NuPage LDS sample buffer; Life Technologies) and pre-stained protein ladder (PageRuler™ 10 to 180 kDa; Thermofisher Scientific). Gels were blotted onto nitrocellulose membranes using the Trans-Blot Turbo device (BioRad) and probed using 1 in 1000 dilution of NRF2 (Cell Signaling Technology; 12721), beta-actin (Cell Signaling Technology; 4967), Phospho-Histone H2A.X (Ser139) (20E3) Rabbit mAb #9718, and TRAIL (anti-human TRAIL; c-terminal; ab42121, Abcam)) with horse-radish peroxidase-conjugated anti-rabbit secondary antibody (Cell Signaling Technology, #7074), using the iBind Flex incubation system (Thermofisher Scientific). ECL developer (ECL Prime, GE) was added to membranes for 5 min and carefully blotted off before imaging (ImageQuant, LAS4000; GE). Band intensity was measured using ImageJ (NIH.gov) with the gel analyse plug-in.

### Animal work

Female mice with severe combined immunodeficiency (NOD-SCID Gamma, strain NOD.Cg-Prkdc scid Il2rgtm1Wjl/SzJ; Charles River, UK) were aged 6–8 weeks and weighed 20–22 g at the time of implantation. Procedures were carried out under the authority of project and personal licences issued by the Home Office, UK, and were approved by local Animal Welfare and Ethical Review Bodies at University College London.

### Cell implantation

A small patch of fur was shaved over the right hand side of the rib cage. Under isoflurane anaesthesia, 1 × 10^5^ human mesothelioma cells (CRL2081, transduced to express firefly luciferase) were injected into the intra-pleural space between the third and fourth rib up from the bottom of the ribcage in 50 μL PBS. Tumour growth was followed up by bioluminescence imaging at 1, 7, 10, 16, and 22 days post-injection, at which point mice were injected intravenously with 1.5 × 10^6 89^Zr-oxine-labelled MSCTRAIL in 200 μL PBS.

### Bioluminescence imaging

Mice were anaesthetised with isofluorane and kept at 37 °C, injected intraperitoneally with 150 mg/kg of D-Luciferin solution, and imaged at 20 min post-injection (for tumour monitoring) and 15 min post-injection (for MSCs) using an IVIS Lumina (Perkin Elmer). Exposure times were optimised to ensure sufficient signal was obtained without saturation. Light output was quantified using ROI analysis and normalised to photons/second/steradian. Cherenkov luminescence from ^89^Zr decay could not be seen above background noise, but was compensated for using images taken prior to luciferin injection with background signal subtracted over the relevant ROI from post-luciferin values.

### PET imaging

Mice were imaged at the stated time points after intravenous injection of ^89^Zr-oxine-labelled MSCTRAIL, using a PET-CT (Mediso NanoScan) interfaced to InterView Fusion software. CT was acquired at 50 kVp with 300-ms exposure and reconstructed in 0.13-mm isotropic voxels. PET data was reconstructed in 5:1 mode using the Tera-Tomo algorithm in 0.4-mm isotropic voxels and analysed using VivoQuant software (InViCro). 3D ROIs were drawn manually around the lungs, liver, kidneys, spleen, and brain, based on CT soft-tissue contrast, and bones were segmented using CT signal thresholding. The percentage of injected dose/organ (%ID/organ) was calculated using decay-corrected ROI values.

### Magnetic resonance imaging

Images were obtained using a 1-T MRI system (ICON; Bruker BioSciences Corporation, Ettlingen, Germany), interfaced to a console running Paravision 5 software (Bruker). A 30-mm mouse body solenoid RF coil (Bruker) was operated on transmit/receive mode. A rectal thermometer and respiratory pad provided physiological monitoring (SA Instruments, New York, USA), with temperature maintained via a water-heated bed. Multi-slice images of the lungs were acquired using a T_2_-RARE sequence, with effective TE of 55.8 ms (echo train length = 6), TR = 1463 ms, 8 averages, 12 slices, field of view of 2.56 × 2.56 cm, 128 × 128 matrix, 1-mm slice thickness, 0.1-mm slice spacing, and respiratory gating. Total scan time was 4 min 5 s.

### Dosimetry estimation

Details of dosimetry calculations can be found in the supplementary information.

## Results

### ^89^Zr-oxine effect on viability of MSCTRAIL is dependent on the amount of labelling activity and time

Umbilical cord tissue-derived mesenchymal stromal cells (uct-MSCs) were transduced using a lentiviral vector encoding TRAIL. TRAIL expression at 95% was confirmed by flow cytometry (Figure S1), and these cells (hereafter referred to as MSCTRAIL) were used for all subsequent experiments except where otherwise stated.

To assess ^89^Zr-oxine cytotoxicity, freshly harvested MSCTRAIL were radiolabelled over a 10-fold range of activity between 1515 and 152 kBq/10^6^ cells (as measured after 3 washes), or sham labelled in PBS alone, or PBS + 3% DMSO (used as a ^89^Zr-oxine vehicle). A 20-min labelling incubation was chosen to facilitate use within the 90-min post-defrosting during which TRAIL-MSCs typically maintain maximum viability when left in the cryopreservant [25]. Labelling efficiency correlated negatively with ^89^Zr-oxine amount (*R*^2^ = 0.804), with the lowest two amounts achieving the highest labelling efficiency with between 29 and 33% retained after 3 washes (Figure S2A), comparable to prior reports using a 30-min incubation [16]. The highest amount showed the earliest effect on viability vs unlabelled cells after 3 days, though all amounts resulted in a significant reduction in cell growth as measured by ATP and NADH metabolism from day 4 onwards (Figure S2B, C). Two-way ANOVA analysis showed significant individual as well as interactive effects from time and amount of ^89^Zr per cell (*p* < 0.001).

**Fig. 1:**
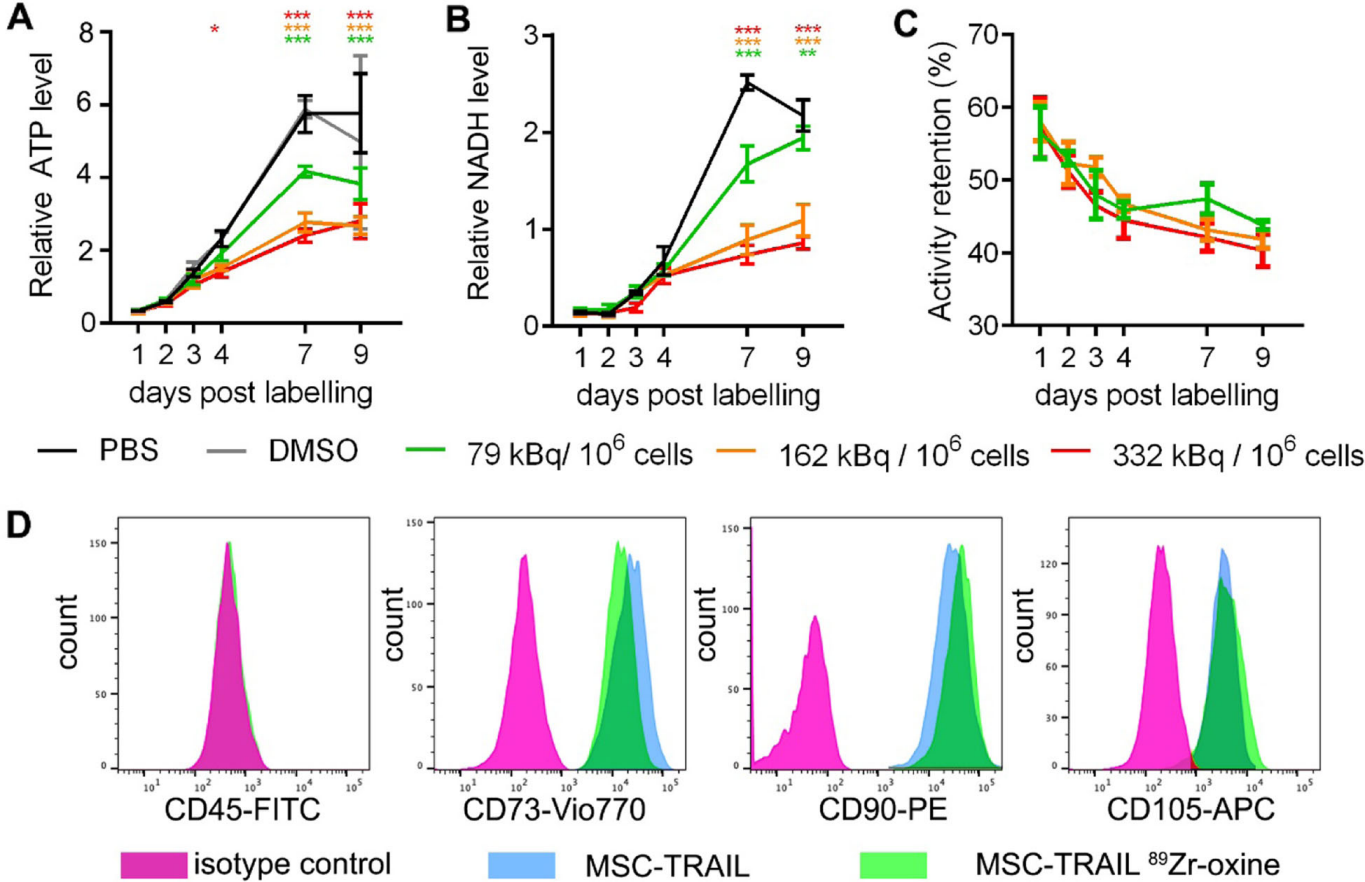
MSCTRAIL show time- and amount-dependent sensitivity to ^89^Zr-oxine labelling but retain MSC phenotype. MSCTRAIL cells were labelled from frozen with ^89^Zr-oxine between 79 and 332 kBq/10^6^. Cells show reduced proliferation with increasing amount as indicated by metabolism of ATP (**a**) and NADH (**b**). Error bars show standard deviation (SD). *p < 0.05, **p < 0.01, ***p < 0.001 2-way ANOVA with Dunnett’s multiple comparisons test vs PBS control. **c** Retention of ^89^Zr oxine decreases over time (*n* = 4). The 2-way ANOVA analysis showed that the majority of variation (67.5%) was due to time (*p* < 0.0001), with amount having a significant (*p* = 0.015) but small effect (3% of variation) on activity retention. **d** MSCTRAIL retain their MSC phenotype (CD45-ve, CD73, CD90, and CD105+ve), post-radiolabelling with ^89^Zr-oxine (amount shown 332 kBq/10^6^ cells)

Cryopreserved MSCTRAIL doses are thawed immediately prior to patient transfusion in the TACTICAL trial [25] and will be radiolabelled between thawing and transfusion for the imaging cohort. For ^89^Zr doses equivalent to 37 to 100 MBq per patient receiving a cell dose of 5 × 10^6^ cells/kg (4 × 10^8^ cells for a patient of 80 Kg), we assessed the effects of labelling MSCTRAIL immediately after defrosting, encompassing the clinical range of 92.5–250 kBq/10^6^ cells. The highest labelling amount (332 kBq/10^6^ cells) showed the earliest effect on ATP metabolism vs sham (PBS)-labelled cells at 4 days post-labelling, though all amounts resulted in a significant reduction in ATP and NADH levels from day 7 (Fig. 1a, b). Time accounted for 74% (ATP) and 68% (NADH) of variation and amount for 9% (ATP) and 13% (NADH) of variation, with significant interaction between labelling amount and time accounting for 11% (ATP) and 17% (NADH) of variation (2-way ANOVA, *p* < 0.0001).

The effect of ^89^Zr amount on activity retention was also investigated (Fig. 1c), with the majority of variation in retention (67%) due to time (2-way ANOVA; *p* < 0.0001), with a small but significant effect from labelling amount (3%, *p* = 0.015). Most of the label loss occurred during the first 24 h after which label loss was slower, with only a further 12% being lost between 24 h and 7 days.

Similar interactive and individual time- and amount-dependent effects on ATP and NADH metabolism were observed in MSCTRAIL labelled directly after harvesting from culture (Figure S3A,B) across a comparable range of ^89^Zr-oxine amounts (72 to 283 kBq/10^6^ cells). Labelling efficiency of frozen MSCTRAIL at 37.7% (SD = 2.2) was comparable to the mean of 43% (SD = 3.6) achieved with cells harvested directly from culture.

Since radiolabelling affects MSCTRAIL cell viability at higher ^89^Zr amounts, we investigated if this was due to cytotoxic or cytostatic effect induced by the radiolabel. Cell cycle analysis of MSCTRAIL labelled directly after thawing (Figure S4) showed changes in cell cycle profile from the 3-day to 7-day post-labelling time points which increased with amount, consistent with metabolic changes (Fig. 1). The proportion of cells in G2/M in the top two radiolabelling amounts doubled compared to the PBS control, suggesting that activation of the DNA damage checkpoint may be responsible for the decreased rate of proliferation at these amounts. At the lower amount (79 kBq/10^6^ cells), cells showed a similar cell cycle profile to control cells at days 3 and 7, consistent with their more similar metabolic profile to unlabelled cells. Increases in apoptotic cell fraction (up to 32% for the higher amount) were only seen at the highest two amounts at day 7, though ~ 7% of cells were still in S phase, consistent with the slow but continuing replication up to day 7 (Fig. 1).

To further assess the effects of radiolabelling, Western blot analysis of cell extracts from 7 days post-labelling was assessed for γ-H2AX upregulation for DNA damage signalling and Nrf2 upregulation for oxidative stress (Figure S5A). Protein upregulation was not detected in radiolabelled compared to control cells at this time point. This suggests that DNA damage has either been repaired in cells by this time point or that it is occurring below the threshold of detection, and that cells are not showing detectable levels of oxidative stress signalling. To confirm the functioning of these homeostatic signalling pathways in cells after radiolabelling, this experiment was repeated using TBHP to induce DNA damage and oxidative stress, which showed upregulation of both proteins in radiolabelled and non-radiolabelled cell populations (Figure S5B).

### Radiolabelling does not affect MSC-specific cell surface marker profile

Flow cytometry was used to evaluate the effect of radiolabelling on MSCTRAIL surface marker phenotype, using the ISCT-approved MSC identification panel of antibodies [26]. Low to high radiolabelled and control (PBS and PBS+DMSO sham labelled) MSCTRAIL showed the expected cell surface marker expression for MSCs (+ve for CD73, CD90, and CD 105; −ve for CD14, CD19, HLA class II, CD34, and CD45), (Fig. 1d and S6). Radiolabelling amounts above and below the range needed for clinical PET imaging of MSCTRAIL are therefore compatible with maintaining an MSC-like cell surface marker phenotype. Cell morphology was also comparable between sham-labelled and radiolabelled MSCs when observed using phase-contrast microscopy at 96 h post-labelling (Figure S7).

### TRAIL expression and therapeutic function is unaffected by radiolabelling

**Fig. 2:**
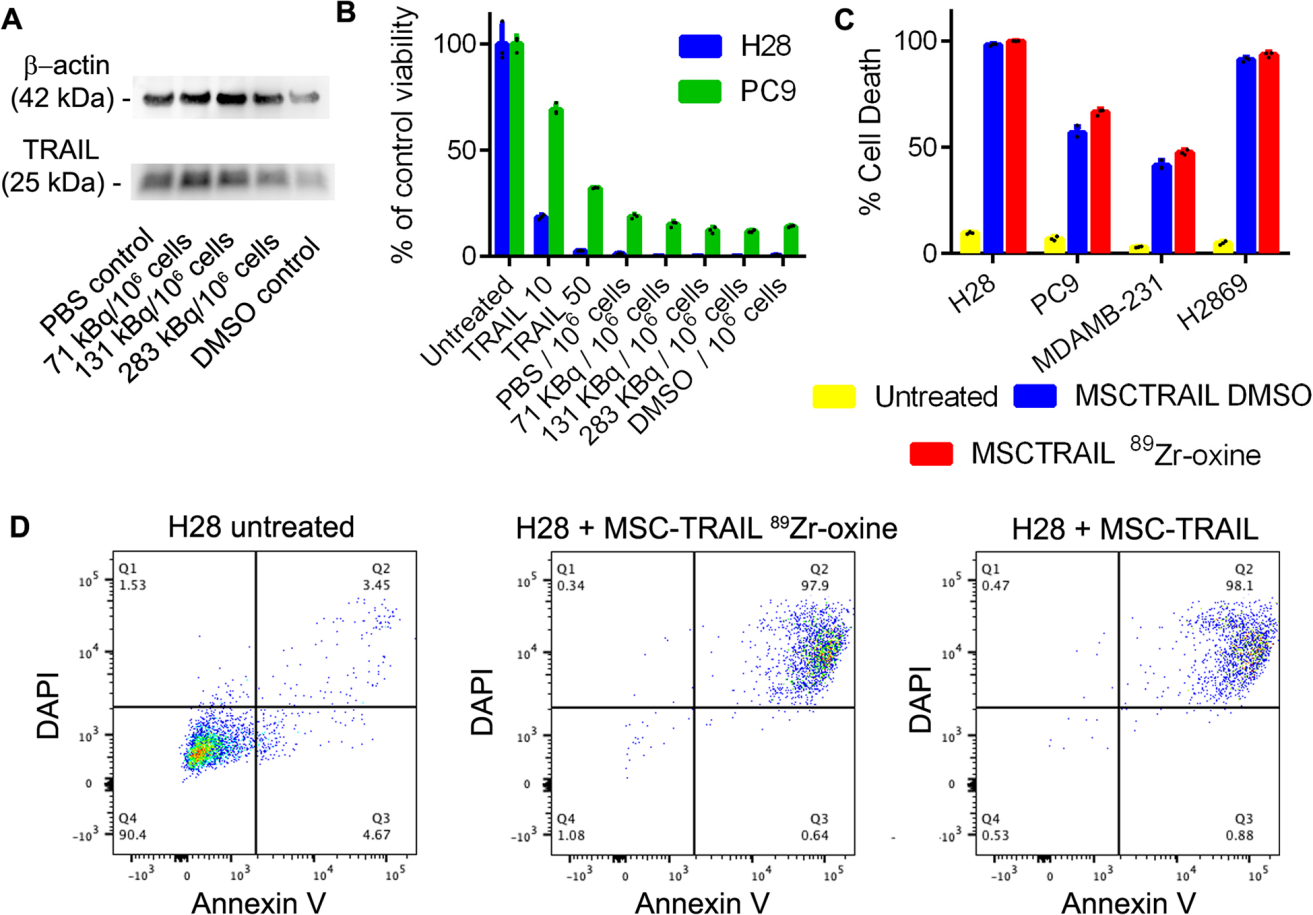
TRAIL expression and therapeutic function is maintained following radiolabelling. **a** Western blot analysis on protein extracts 7 days post-radiolabelling shows maintained TRAIL expression. **b** Viability of two human lung cancer cell lines (NHI-H28 and PC9) was measured using luciferase bioluminescence after treatment with soluble trail at 10 or 50 ng/mL, or co-incubation for 24 h with MSCTRAIL labelled 3 days prior with the indicated amounts, or sham labelled in PBS or PBS with 3% DMSO. Bars show the mean of 4 populations; error bars show SD. **c** Apoptosis of four cancer cell lines untreated or after incubation with MSCTRAIL (sham labelled in PBS + 3% DMSO) or MSCTRAIL radiolabelled with ^89^Zr-oxine at 332 kBq/10^6^ cells, measured using Annexin V staining and flow cytometry. Error bars show SD, *n* = 3. **d** Representative FACS plots from **c**, showing apoptosis of H28 cells following incubation with control or radiolabelled MSCTRAIL

To assess the effect of ^89^Zr-oxine on therapeutic capacity, maintained TRAIL expression on MSCs was confirmed 7 days post-labelling with Western blot (Fig. 2a). Labelled and unlabelled MSCTRAIL were co-cultured with luciferase-expressing TRAIL-sensitive (NCI-H28, human lung mesothelioma) and partially TRAIL-resistant (PC9; human lung adenocarcinoma) cancer cells (Fig. 2b). Reduction in viable cancer cell population was measured as light output following the addition of luciferin and compared to untreated control populations. Soluble recombinant TRAIL at 10 and 50 ng/mL was used as a positive control. Population reduction of viable cancer cells was further confirmed by light microscopy (Figure S8A). Radiolabelled MSCTRAIL maintained apoptosis-inducing ability against both cancer cells lines (Fig. 2b). An orthogonal cell death assay by Annexin V/DAPI flow cytometry in four cancer cell lines (H28, PC9, MDAMB-231, H2869) also confirmed maintained induction of apoptosis by MSCTRAIL post-radiolabelling, giving equivalent results (Fig. 2c, d, S8B).

### In vivo TRAIL-MSC tracking in a mouse model of lung mesothelioma

**Fig. 3:**
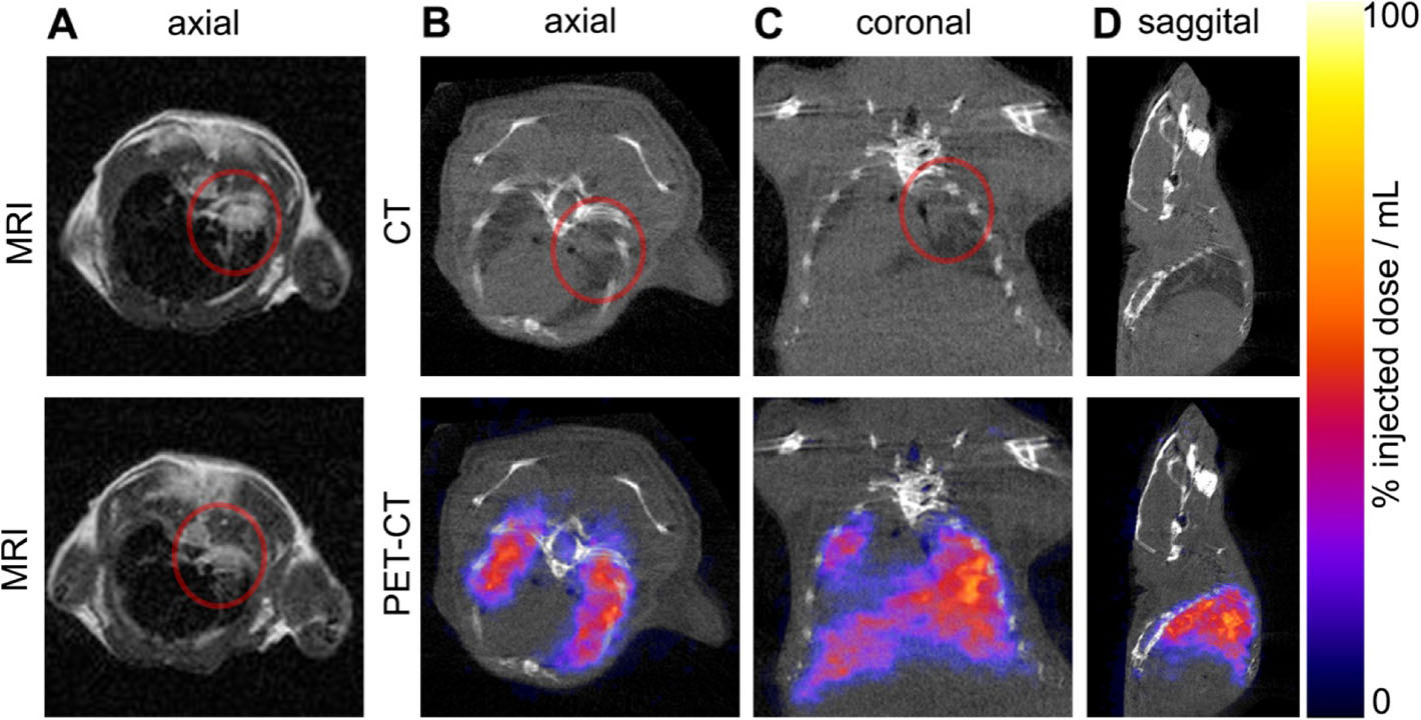
Tumour visualisation with MRI and CT and location of ^89^Zr-oxine-labelled MSCTRAIL visualised with PET at 2 days post-transplantation. Lung tumours detected with **a** axial T_2_-weighted magnetic resonance imaging (T_2_ RARE TE = 55 ms, respiratory gated) showing two consecutive 1-mm-thick slices through the lung tumour (shown in the red circle) at 15 days post-implantation **b** axial, **c** coronal, and **d** sagittal CT slices, with corresponding PET overlay showing the location of ^89^Zr-oxine-labelled MSCTRAIL in the lungs and liver

Immunocompromised mice were implanted intra-pleurally in the right hand side with a human mesothelioma cell line (luciferase-transduced CRL-2081), and tumour growth was followed up using bioluminescence imaging (Figure S9). T_2_-weighted magnetic resonance imaging and CT showed localisation of the tumour to the right lung (Fig. 3a to c) at 15 days post-implantation. MSCTRAIL were then thawed, radiolabelled (311 kBq/10^6^ cells), and injected intravenously (1.5 × 10^6^ cells). PET-CT showed ^89^Zr signal throughout the lung, including within the area containing the tumour (Fig. 3b to d).

**Fig. 4:**
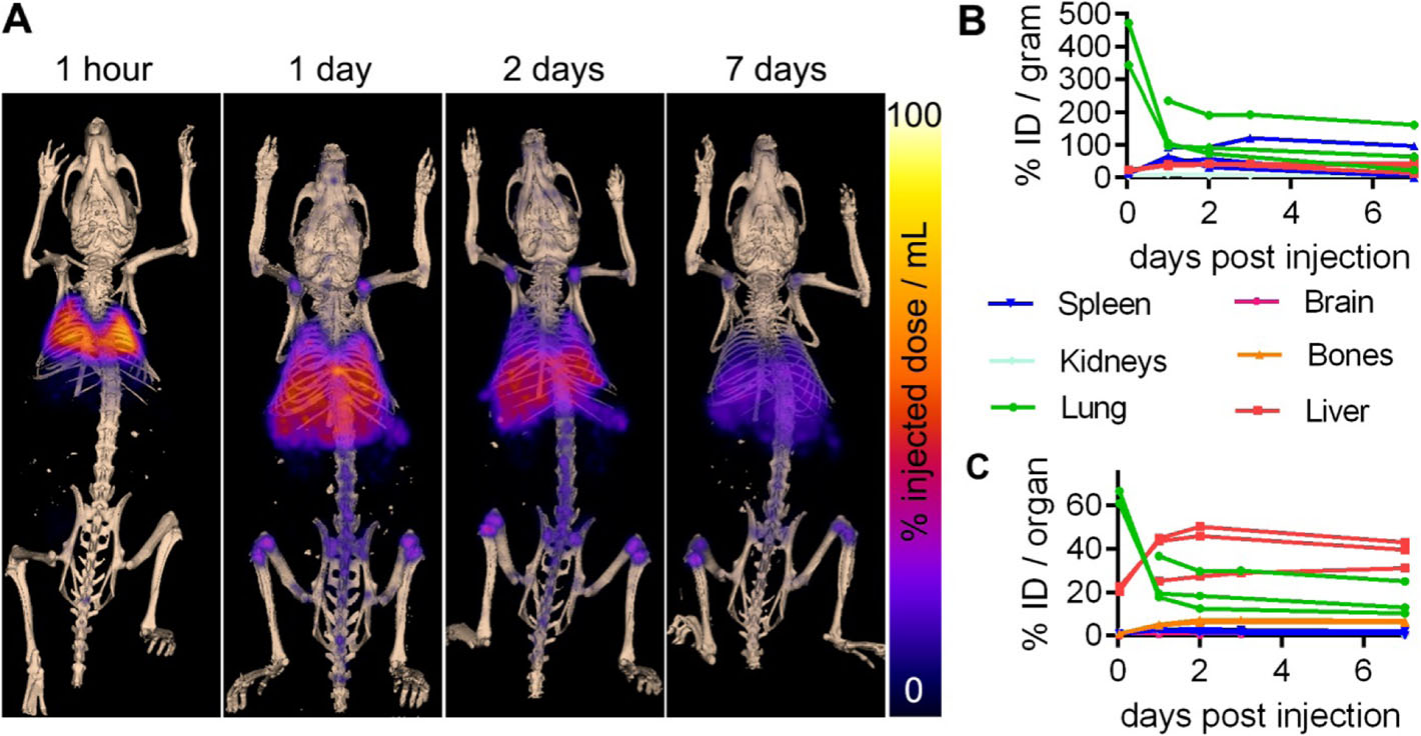
Whole-body biodistribution of ^89^Zr-oxine-labelled MSCTRAIL followed up to 1 week post-implantation. **a** Maximum intensity-projection PET showing ^89^Zr-oxine-labelled MSCTRAIL overlaid on 3D-rendered bone CT at the indicated time points after intravenous injection. The indicated organs and bones were segmented using CT data and radioactivity quantified from PET data, giving **b** % injected dose (ID) per gram, using the wet weight of tissue from each animal (*n* = 3) following dissection at day 10 post-MSCTRAIL injection, and **c** % ID per organ

PET-CT at 1 h and 1, 2, and 7 days post-injection enabled visualisation and quantification of MSCTRAIL biodistribution dynamics (Fig. 4). The majority of signal (60%) was found in the lung at 1 h before decreasing, while liver signal increased. From 1 to 7 days post-injection, the proportion of the ^89^Zr signal in the lung fell further from 24.6% (± 10.4% SD) to 16.0% (± 7.9% SD). Uptake in the spleen was also visible and peaked at 2.6% of the total injected dose (± 0.64% SD) at 48 h.

At 10 days post-MSCTRAIL injection, organs were removed and weighed, and ^89^Zr activity was measured. Activity per organ was normalised to organ weight and decay-corrected (Figure S10 and Table S1). Consistent with PET-CT and ROI analysis, high amounts of activity in the lung and liver were found, with the lung having the most activity per gram (78% ID per gram ± 26%) followed by the liver (32.8% ID per gram ± 8.6%), though the liver had a higher absolute percentage of the injected dose (29.4% ± 11.5%) compared to the lungs (12.4% ± 4.3%). Activity uptake in the spleen was also still high at 10 days post-injection (43.9% ± 5.3% per gram), consistent with its visibility in the PET-CT scans (Fig. 4a); however, in absolute terms, this represented just 1.6% ± 0.5% of the total injected activity.

### Correlation of PET signal with viable cell location

**Fig. 5:**
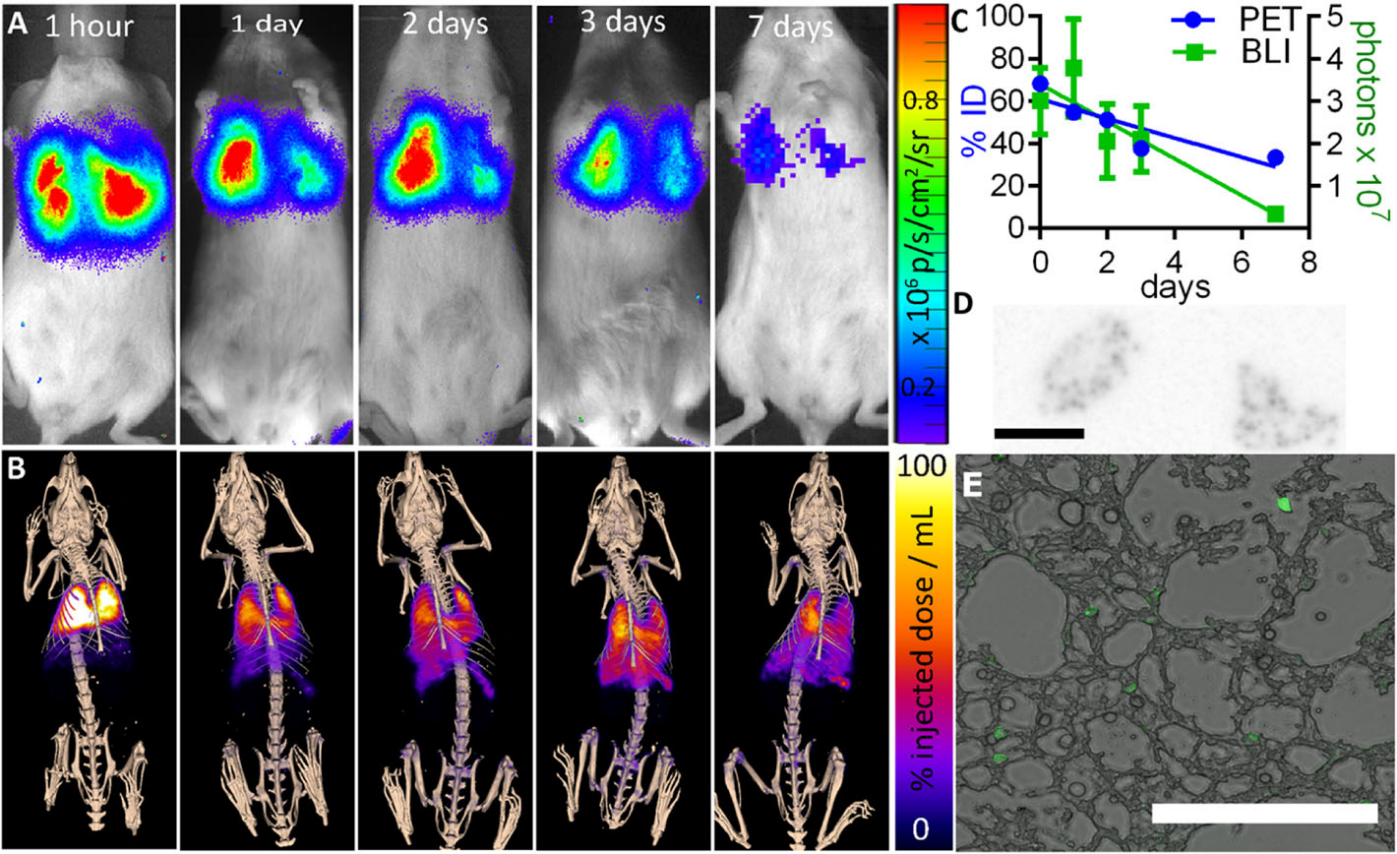
Comparison of viable MSC location to PET signal. Intravenously injected ^89^Zr-oxine-labelled uct-MSCs expressing luciferase and ZsGreen were tracked over 7 days using **a** bioluminescence imaging and **b** PET-CT imaging. **c** ROI analysis of PET and BLI data showed a comparable decrease in %ID and photons in the lungs over time (*n* = 3, error bars SD). At 7 days post-injection, lungs were removed and cryosectioned, before imaging with **d** autoradiography (scale bar 5 mm) and **e** fluorescence microscopy (scale bar 200 μm), to confirm the presence of ZsGreen-expressing cells and ^89^Zr signal within the lungs

If ^89^Zr-oxine labelling is to be of practical use in tracking cell biodistribution, PET signal must correlate with viable cell location over biologically relevant timeframes. To evaluate this, uct-MSCs were transduced to express luciferase and the green fluorescent protein ZsGreen to enable independent viability and/or location confirmation with in vivo bioluminescence imaging (BLI) and ex vivo fluorescence microscopy. MSCs expressing ZsGreen luciferase were then radiolabelled with ^89^Zr-oxine (412 kBq/10^6^ cells), injected intravenously, and imaged with PET-CT and BLI for 7 days. This showed correlation of radiolabel with viable cell location, with an initial predominance of signal in the lung which decreased over time on PET and BLI (Fig. 5a–c). Organs were then removed, cryosectioned, and imaged with fluorescence microscopy and autoradiography (Fig. 5d, e), together with DAPI staining (Figure S11), confirming the presence of the transplanted ZsGreen-expressing uct-MSCs in the lungs.

### Regions of background ^89^Zr uptake

Despite a significant fraction of injected radioactivity being measured in the liver, spleen, and bones from 1 day post-injection onward, and at the end time point, no signal was detected in these organs with in vivo, *or* ex vivo bioluminescence (Figure S12A,B), suggesting either dissociation of the label from MSCs, or the uptake of labelled but dead MSCs or debris derived from these. Consistent with this interpretation, examination of tissue sections with fluorescence microscopy did suggest the presence of debris from ZsGreen-expressing cells (S12D,E), which was not visible in sections taken from control animals not receiving MSCs (S13). We also saw liver and spleen uptake of intravenously injected heat-inactivated MSCs seen with PET-CT, which supports the role of the liver and spleen in taking up labelled dead cells (S14), consistent with previous reports [27]. An additional likely source of liver and spleen signal is the ^89^Zr lost from labelled MSCs over time (Fig. 1c). Zirconium has been shown to have a strong affinity for phosphate, and ^89^Zr-phosphate has been shown to have high uptake in the liver and spleen, but not in the lungs. Free zirconium species such as its chloride or weakly chelated forms have also been shown to be taken up by the bone [28].

### Human dosimetry estimates

Human dosimetry estimates were calculated with OLINDA software [29] using mouse to human extrapolations according to Stabin [30] and the preclinical in vivo region of interest analysis data and ex vivo biodistribution data (see Table S2 to S4). For an injected activity of 37 MBq, this gave mean effective dose estimates for male and female patients of 32.2 and 41.4 mSv, respectively. For 100 MBq per patient, this corresponds to an effective dose of 87.1 and 111.8 mSv for male and female patients, respectively. The organ-specific dose is estimated to be highest in the lungs (5.09, 6.58 mSv/MBq), spleen (2.12, 2.57 mSv/MBq), and liver (1.86, 2.39 mSv/MBq) for male and female patients, respectively.

## Discussion

Many factors potentially contribute to the complexity of cell behaviour and cell/host interactions including cell source and pre-processing, injection route, patient age, immune system, co-morbidities, genetics, life history, and microbiota [31–33]. Without assessing cell biodistribution in patients using cell tracking techniques, it remains difficult to evaluate the effect of these variables on cell behaviour and on the failure of many emerging cell-based therapies [34].

To support integration of ^89^Zr-oxine cell tracking into the TACTICAL trial, we have shown that TRAIL-expressing umbilical cord tissue-derived MSCs (MSCTRAIL) can be tracked non-invasively to the lungs in a preclinical lung cancer model up to 7 days post-injection. PET signal corresponded to viable cell signal from bioluminescence imaging, increasing confidence in the reliability of this technique. This lung uptake and retention of MSCs following intravenous injection is also consistent with previous reports in small [27, 35, 36] and large [37, 38] animal imaging studies, as well as patients [39]. Though intravenously injected MSCs have also been shown to subsequently migrate from the lungs to tumours or other injured or healthy organs such as the heart and bone marrow [14, 37], this finding has not been universal. Other studies have shown that MSCs sometimes remained trapped in the lungs after IV injection, where they rapidly lose viability before clearance of labelled cell debris to the liver and spleen [14, 27]. This variability between findings can variously be attributed to a range of complex interacting factors that differ between these studies, including source, species, dose and preparation of MSCs, species of animal model, and its disease state [14]. Though the results here are not enough to attribute the lung delivery and retention of MSCs to a specific tumour homing effect, they nevertheless support the intravenous route as an effective means of delivering MSCs to the lung.

Here, both BLI and PET-CT showed the loss of MSCs in the lung over the course of the week, suggesting that repeat MSCTRAIL dosing will be necessary (3 cycles of MSCTRAIL doses are given at 21-day intervals in TACTICAL). However, MSC-host interactions are likely to differ between these preclinical results where MSCs are xenogeneic to the host and the clinical scenario where they are allogenic. Inevitably, some signal was also found in areas not associated with live cells, (i.e. the liver, spleen, and bones), though these were consistent with known uptake areas of free zirconium [28] and MSC-derived debris [27]. Labelling was achieved from frozen cell stocks with relevant amounts of ^89^Zr-oxine within 45 min—below the 90 min over which frozen MSCTRAIL retain optimal viability post-thawing [25], suiting this approach to translation.

PET imaging of ^89^Zr antibodies has been reported up to 5 days post-injection with 37 MBq/patient [40, 41] and up to 6 days using 70 to 75 MBq/patient [42, 43]. To achieve 37 MBq/patient receiving 5 × 10^6^ MSCTRAIL cells/kg (i.e. 4 × 10^8^ cells for an 80-kg patient), this equates to ^89^Zr-oxine labelling at 92.5 kBq/10^6^ cells—towards the lower range of amounts assessed here. Even at higher amounts, TRAIL expression, therapeutic efficacy, and MSC cell surface markers were retained. On the other hand, decreased proliferation rates were seen by days 4 to 7, indicating that some caution needs to be taken in optimising labelling amounts for cell therapies in which proliferation is desired.

Previous PET imaging studies have used ^18^F-based labelling techniques for tracking cell-based therapies in patients. For example, ^18^F-FDG has been used clinically to track homing of bone marrow cells to the infarcted myocardium [31] and islet cell delivery to the liver [44]. An advantage of this technique vs ^89^Zr-oxine labelling is that ^18^F-FDG is widely available, and the short half-life (110 min) reduces patient radiation exposure. However, the short ^18^F half-life also prevents tracking of cells over longer and possibly more interesting biological timeframes. On the other hand, the 3-day half-life of ^89^Zr can permit observation of labelled cells over a week as shown here, providing more information on their longer term retention, clearance, and migration.

Currently, bone marrow-derived MSCs (bm-MSCs) predominate among approved MSC-based cell therapy products, but their use in clinical trials relative to other MSC sources has more than halved since their peak [45, 46]. Conversely, the allogenic use of umbilical cord tissue-derived MSCs for cell therapy, as in TACTICAL, is increasing due to various advantages including hypo-immunogenicity [45, 47, 48]. Sourcing of cord donor material is non-invasive, unlike harvesting the bone marrow, while production scale-up for cell therapy is eased by their delayed senescence onset, lack of contact inhibition enabling higher harvest densities, and faster proliferation rate [47, 48].

Aside from the present study on umbilical cord tissue-derived MSCs, ^89^Zr-oxine labelling and PET imaging have also been demonstrated with T cells, dendritic cells, and bone marrow cells [16, 17, 21, 23]. Together, these studies illustrate the benefits of ^89^Zr-oxine labelling: fast implementation, non-invasive tracking over a week post-injection, and interpretation of images uncomplicated by the degree of background signal given by the systemically administered tracers used with nuclear reporter-gene systems [49]. Though a modest amount of radiolabel is inevitably lost from cells, the areas that take up free ^89^Zr, including the liver, spleen, and bone, have been well characterised [28]. While this knowledge reduces ambiguity to some degree during analysis, it would also mask the detection of ^89^Zr-labelled cells in these tissues, limiting practical usage to tracking delivery to other tissues such as the lung. The inability to determine whether signal comes from labelled cells or from endogenous cells that have taken up leached signal, or labelled debris from dead cells, is a problem common to all direct cell-labelling techniques. This must therefore be considered during interpretation of results, along with several further limitations. Firstly, the amounts of ^89^Zr required for tracking cells may reduce proliferation, and this effect varies between cell types and time post-labelling. Secondly, though a good correlation between PET signal and viable cell location was shown here using bioluminescence imaging, ^89^Zr-oxine signal does not directly indicate cell viability or proliferation. Hence, for rapidly and non-uniformly expanding cell populations, genetic labelling strategies may be more appropriate [49, 50].

## Conclusion

This study shows that umbilical cord tissue-derived MSCs, and MSCTRAIL derived from these, can be radiolabelled with ^89^Zr-oxine. Phenotype and therapeutic effects post-labelling were retained, and cells could be tracked in vivo up to 7 days using PET. This supports the feasibility of a first-in-man ^89^Zr-oxine cell-labelling arm in phase II of the TACTICAL clinical trial with relatively low labelling amounts, where it should lead to a better understanding of this experimental cell therapy, its whole-body biodistribution dynamics, and inter-patient variability.

## Supplementary information

**Additional file 1.**

## Data Availability

The data and materials that support the findings of this study are available from the corresponding author upon reasonable request.

## Abbreviations

BLI: Bioluminescence imaging
CT: X-ray computed tomography
^18^F-FDG-: 18F-fluorodeoxyglucose
MRI: Magnetic resonance imaging
MSCTRAIL: MSC expressing TRAIL
PBS: Phosphate-buffered saline
PET: Positron emission tomography
RCY: Radiochemical yield
ROI: Region of interest
TACTICAL: Targeted stem cells expressing TRAIL as therapy for lung cancer
TBHP: tert-Butyl hydroperoxide
TRAIL: TNF-related apoptosis-inducing ligand
ucMSC: Umbilical cord-derived MSC
