# Supplementary material for: Lung delivery of MSCs expressing anti-cancer protein TRAIL visualised with 89Zr-oxine PET-CT
Source: Stem Cell Res Ther. 2020 Jun 26;11:256. doi: 10.1186/s13287-020-01770-z (PMC7318529; doi:10.1186/s13287-020-01770-z)
Supplement: Supplementary file 1 — Additional file 1. [file 13287_2020_1770_MOESM1_ESM.docx]

**Supplementary Methods**

**Cell Cycle Analysis**

Populations of MSCTRAIL labelled from frozen were analysed at 72 hours and 7 days post radiolabelling using a Chemometec NC-3000 benchtop image cytometer, using the DAPI-based cell cycle assay according to the manufacturer’s instructions.

**Supplementary figures**


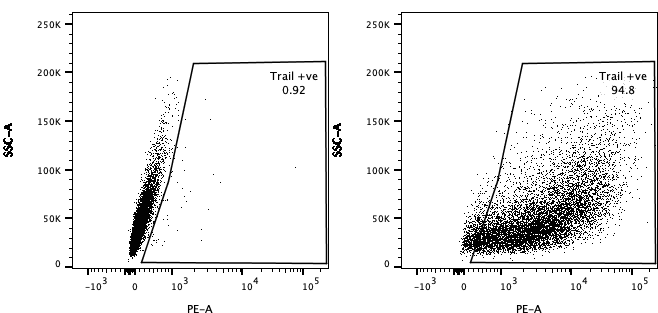


**Figure S1. Flowcytometry analysis of** TRAIL expression of lentivirally-transduced cord-derived MSCs, showing 94.8% positive expression of TRAIL.


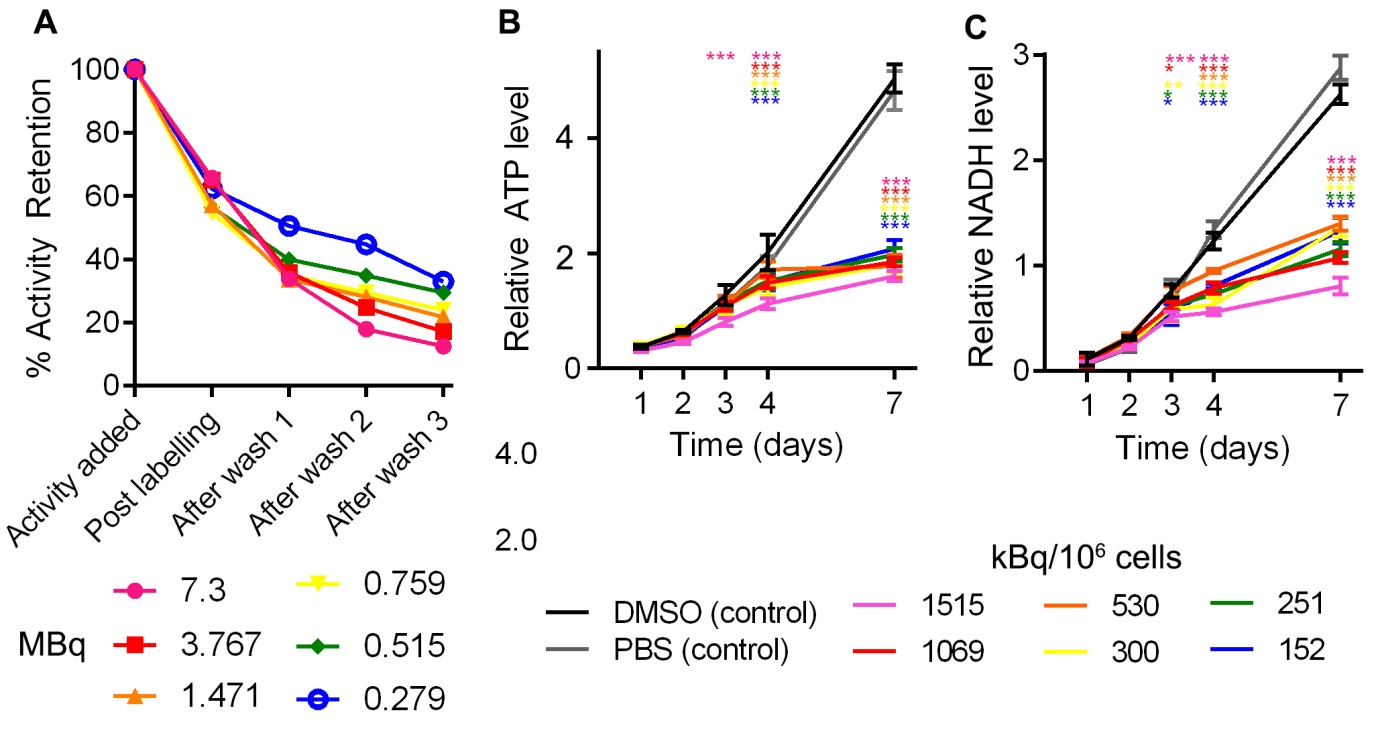


**Supplementary figure 2.** (**A)** 1.5 x 10^6^ cells were labelled in a final volume of 300uL, containing the indicated initial amounts of ^89^Zr-oxine (7.3 MBq to 0.279 MBq) and 3% DMSO. Three washes were found to be sufficient to wash the majority of the unbound activity from the cells. Final Labelling efficiency correlates negatively with the initial amount of ^89^Zr-oxine added to cells (R^2^=0.804). Effect of radiolabelling on cell metabolism was measured using assays for ATP **(B)** and NADH **(C)**. Metabolism of labelled cell populations began to diverge from sham labelled (DMSO and PBS controls) populations at 3 days post labelling. Results were analysed using a 2-Way ANOVA with Dunnett’s multiple comparisons test comparing each population at a given time point against the PBS sham-labelled population * p<0.05 ** p<0.01*** p<0.001. No difference between the PBS and DMSO sham labelled populations was found at any time point. Dosing accounted for 10.4% (ATP) and 10.5% (NADH) of variation (p<0.001), Time for 62.6 (ATP) 69.8 (XTT) (p<0.001), with a significant interaction between time and dose accounting for 25.8% (ATP) and 18.8% (NADH) of variance (p<0.001). Points show the mean of 3 individually seeded populations, error bars show standard deviation (SD).

**
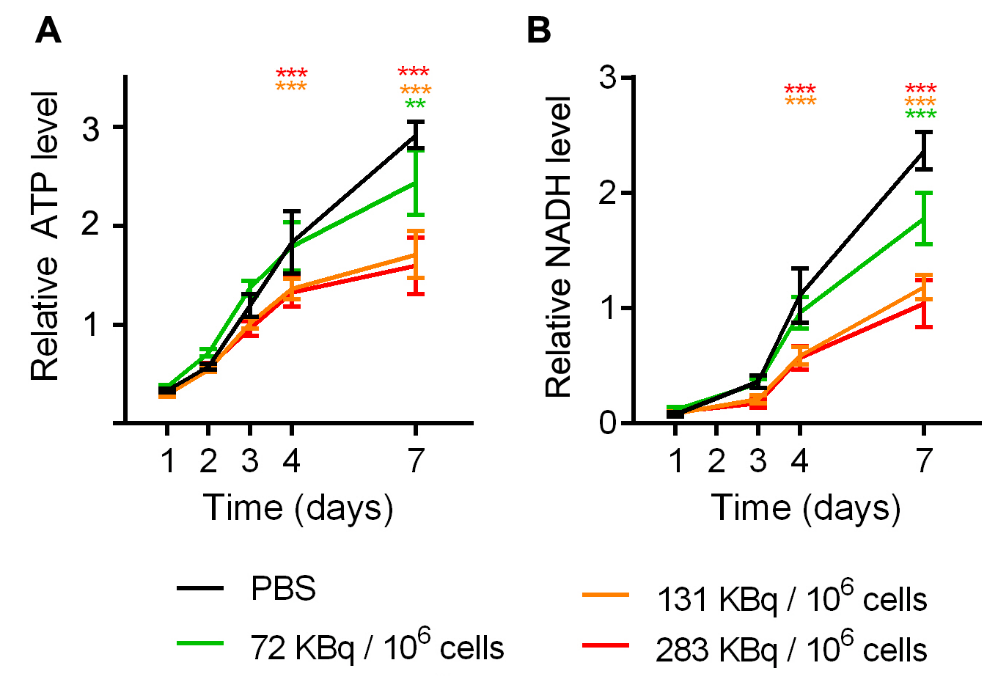

Supplementary Figure 3.** **MSCTRAIL show time and dose-dependent sensitivity to ^89^Zr-oxine labelling.** MSCTRAIL were labelled following harvesting from culture with doses between 283 kBq/10^6^ cells and 72 kBq/10^6^ cells. Cells show reduced proliferation with increasing dose as indicated by metabolism of **(A)** ATP and **(B)** NADH. Dose, time, and dose-time interaction showed significant effect on variation (2-Way ANOVA; p<0.001). Time accounted for 74 % (ATP) and 83.7 % (NADH) of variation, dose for 9% (ATP) and 7% (NADH), with significant time/dose interaction of 5.6 % (ATP) and 7 % (NADH). Here the lowest dose (72 kBq/106 cells) showed a reduced effect on metabolism compared to the higher doses, with no significant difference in ATP or NADH metabolism compared to control cells until 7 days post labelling (p<0.01), whereas higher doses showed a difference by day 4 (p<0.01), Dunnett’s multiple comparisons test.

**
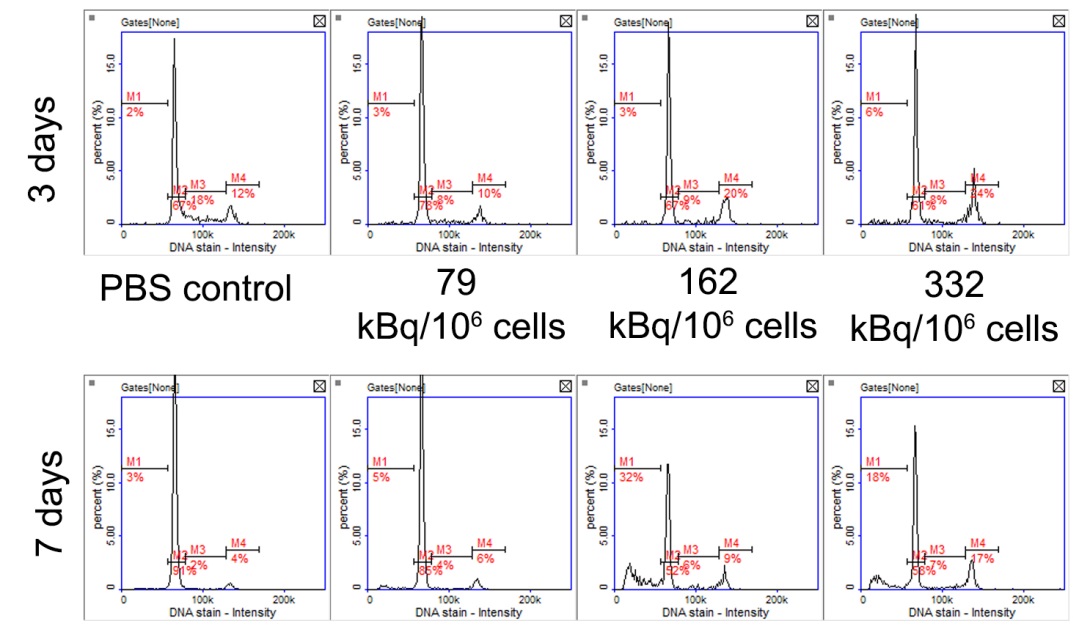
Supplementary Figure 4.** Cell cycle analysis was performed on populations of 10,000 cells per sample for the indicated doses and time points. At 3 days post dosing, the percentage of cells in S phase halved from 18% for the PBS control to 8% and 9% for the radiolabelled conditions. At the same time, the proportion of cells in G2 (the DNA damage checkpoint) doubled from 12% in the control condition to 20 and 24% in the top two radioactive doses. At 7 days post labelling, there was a large increase in the proportion of apoptotic cells in the top two radiolabelling doses, which was not seen at 3 days post labelling.


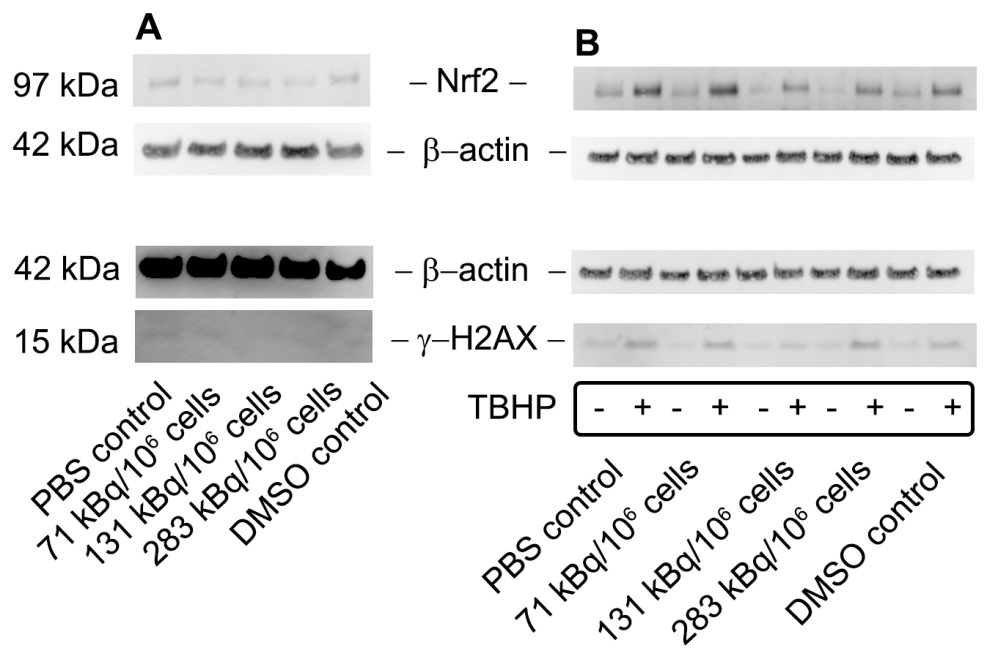
**Supplementary Figure 5.** Western Blot analysis shows comparable expression of cell stress-associated protein Nrf2 and DNA damage-associated protein γ-H2AX in ^89^Zr-oxine labelled and unlabelled control cells at **A.** 7 days post radiolabelling. **B.** Treatment with 200 μM TBHP (tert-butyl-hydroperoxide) for 1 hour induces reactive oxygen stress and DNA damage, and upregulates Nrf2 and γ-H2AX expression in control and radiolabelled cell populations at 3 weeks post radiolabelling, confirming the sensitivity of these signalling pathways.

**
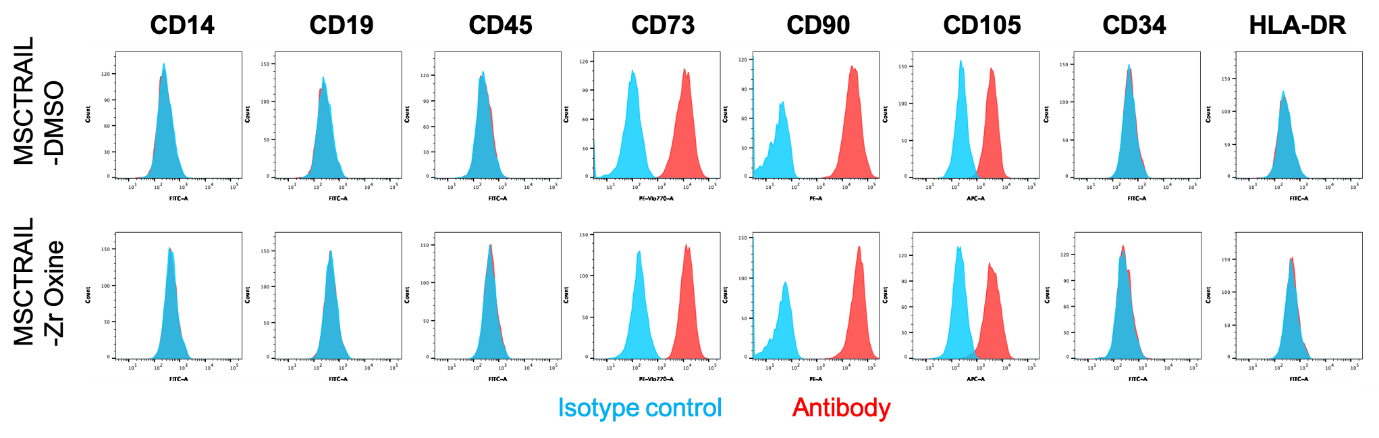
**

**Supplementary figure 6.** Cell surface marker phenotype was assessed using the ISCT-approved panel of MSC identification antibodies at 3 weeks post radiolabelling. Control (DMSO+PBS sham labelled) and radiolabelled (332 kBq/10^6^ cells) populations show positive staining for CD73, CD90, and CD 105, and negative staining for CD14, CD19, CD45, CD34 and HLA DR. PBS only (control) and lower radiolabelling doses (79, 162 kBq/10^6^ cells) gave comparable results (not shown).


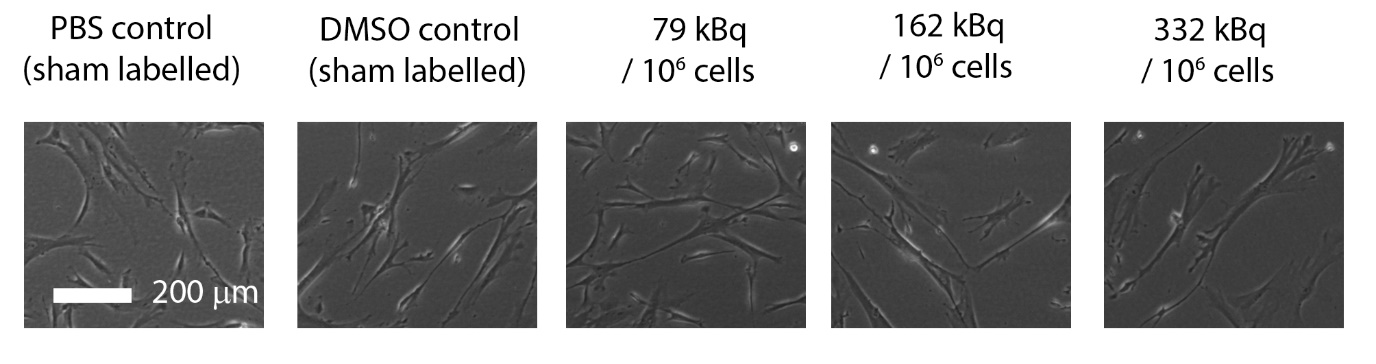
**Supplementary figure 7.** Phase contrast microscopy showing MSCTRAIL 96 hours post labelling with PBS (sham labelled control), PBS with 3% DMSO (sham labelled control), or 79 to 332 kBq/10^6^ cells of ^89^Zr-oxine.

**
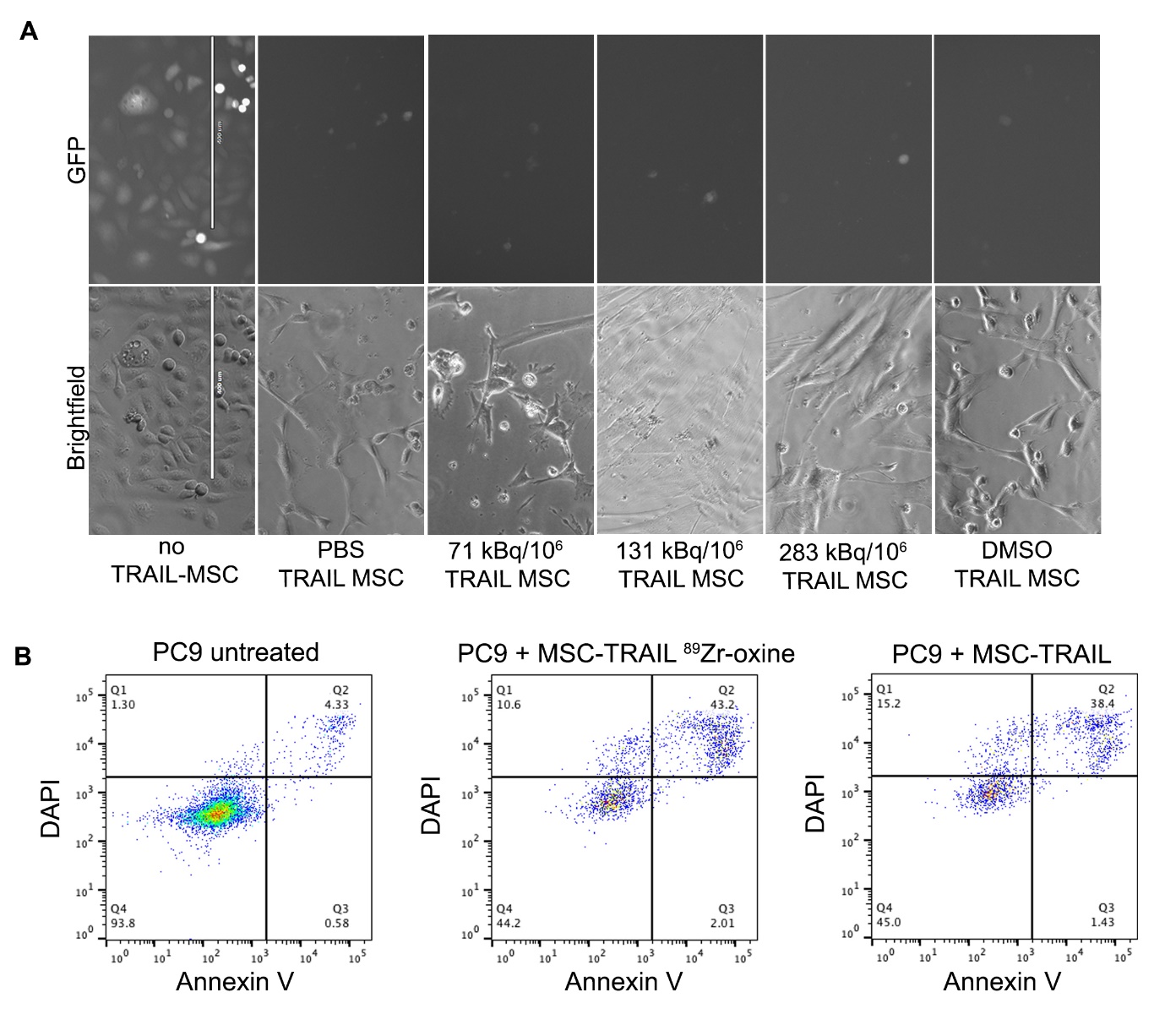
**

**Supplementary figure 8. MSCTRAIL cells retain ability to induce apoptosis in cancer cells post radiolabelling. A.** H28 Cells maintain viability in the absence of treatment (no MSCTRAIL), as seen by the presence of green fluorescent cells. Following treatment with sham dosed (PBS and DMSO MSCTRAIL), or radiolabelled MSCs at the indicated doses, GFP-expressing H28 cells are no longer seen and only MSCs are visible. Scale bars are 400 μm. **B.** Apoptosis is induced in PC9 cells following incubation with MSCTRAIL or radiolabelled MSCTRAIL (332 kBq/10^6^ cells).

**
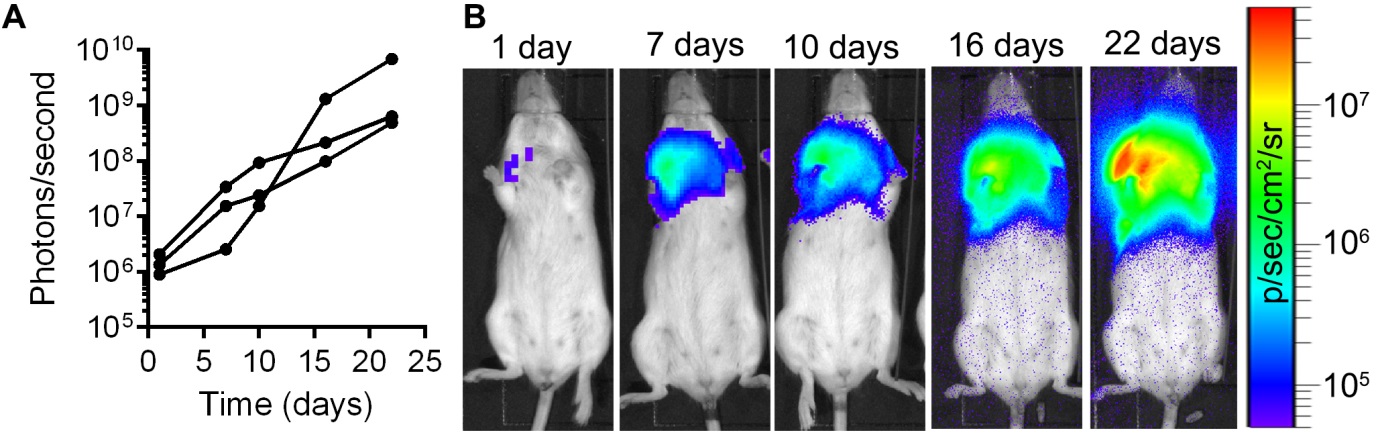
Supplementary figure 9.** Growth of luciferase-expressing human lung mesothelioma cells (CRL2081) in mouse lungs was monitored using bioluminescence imaging at 20 minutes post injection of D-luciferin. **A.** Tumour growth represented as bioluminescent light output from a region of interest drawn over the lung area. Points show the light output from each time point post tumour implantation for three individual mice. **B.** Bioluminescence images from a representative mouse showing lung tumour growth up to 22 days post implantation. Light output intensity is indicated as colour heat-map overlaid on a bright-field image of the mouse.

**
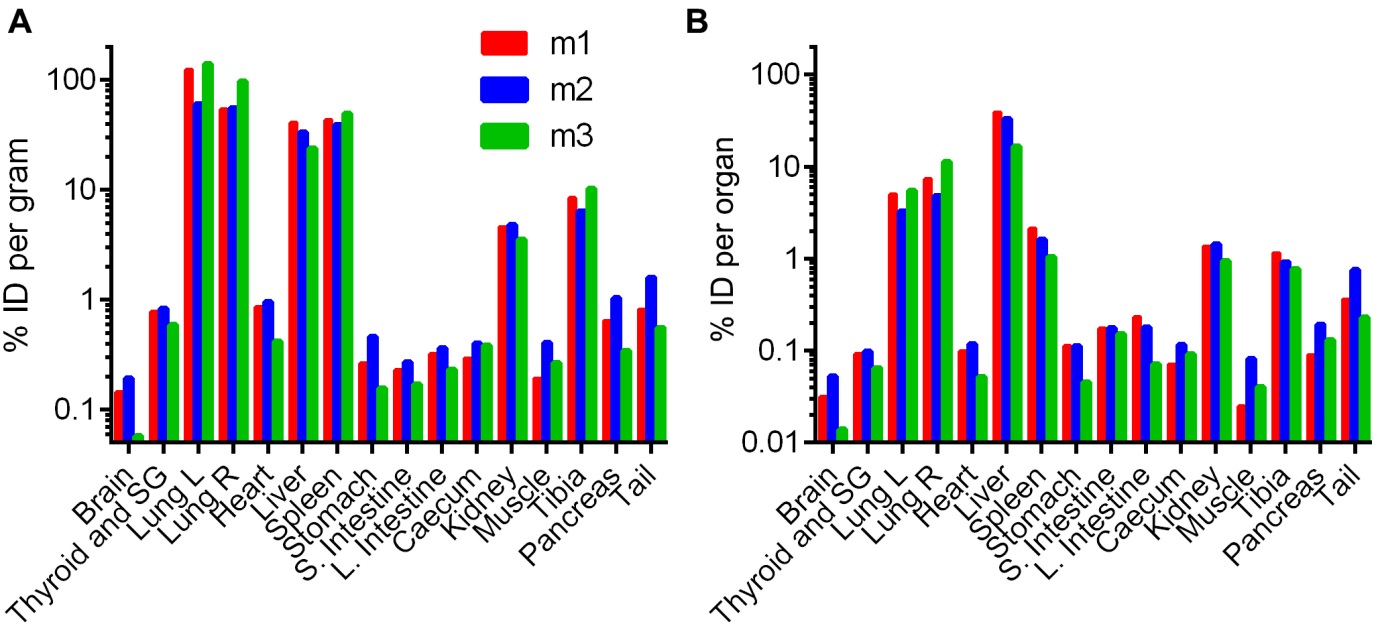
**

**Supplementary figure 10.** Biodistribution data at 10 days post ^89^Zr-oxine labelled MSCTRAIL injection showing decay-corrected ^89^Zr activity as % Injected dose **A.** per gram of wet tissue weight, and **B.** per organ for each of 3 mice. Note that the tibia does not represent the total amount of activity in the bone.


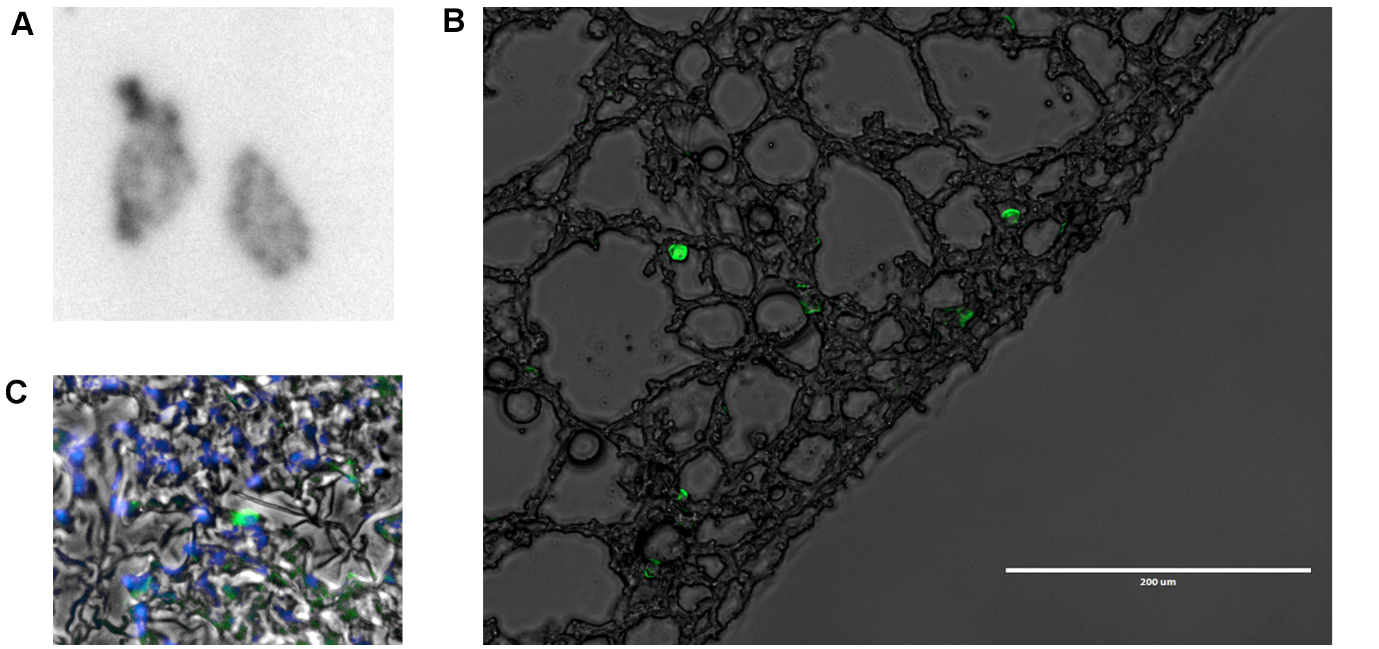


**Supplementary figure 11. A.** Autoradiography of cryosectioned (20 µm) lung tissue taken at 7 days post injection of ^89^Zr-oxine labelled cord-derived MSCs expressing ZsGreen and Luciferase **B.** Green fluorescence microscopy (ex. 470 nm; em. 510 nm) showing ZsGreen-expressing cord-derived MSCs overlaid onto phase contrast image of lung tissue shown in A. **C.** DAPI fluorescence showing nuclei, overlaid with green fluorescence showing ZsGreen-expressing cord-derived MSCs, overlaid onto phase contrast image of lung tissue shown in A.


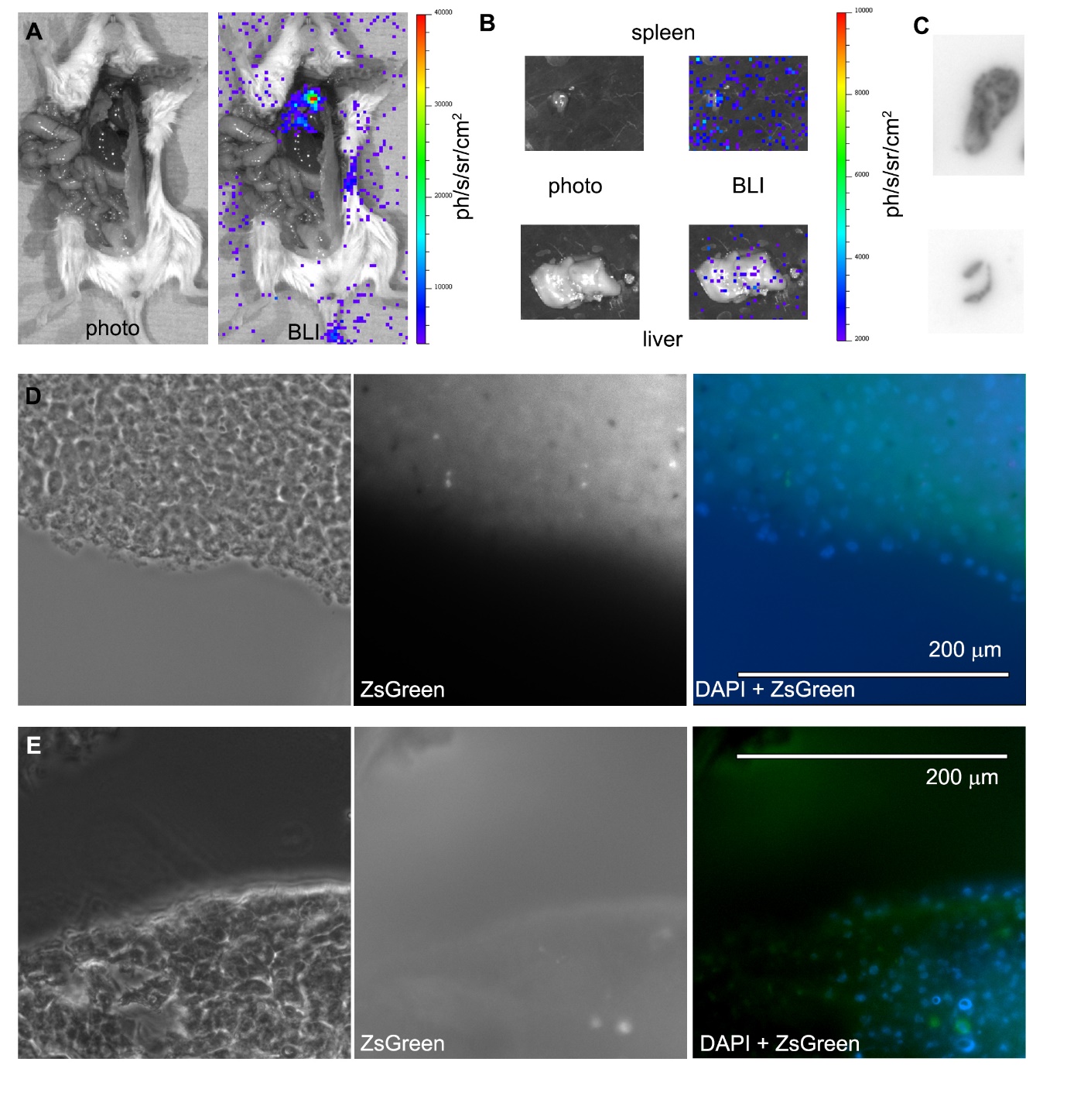


**Supplementary Figure 12.** Ex vivo bioluminescence imaging showing **A.** opened mouse chest and abdominal cavity with signal visible in the lungs, and **B.** dissected liver and spleen showing background levels of signal. **C.** Autoradiography on cryosections of liver and spleen. Light and fluorescence microscopy showing slices of **D.** Liver, and **E.** spleen, showing nuclei stained with DAPI, and ZsGreen fluorescence showing possible debris from injected MSCs (ex 470 nm, em 510 nm).


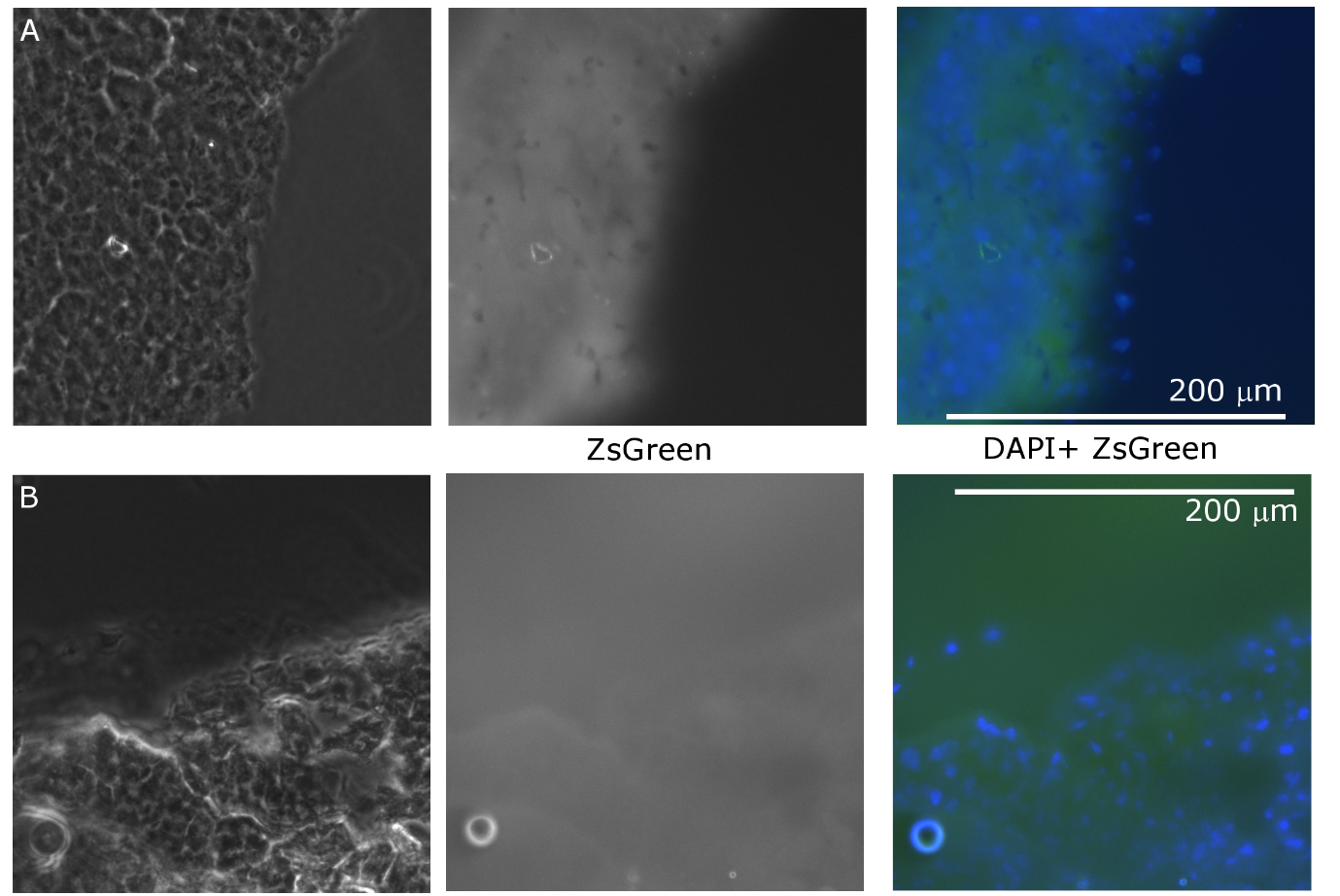


**Supplementary figure 13.** Microscopy images of a representative **A.** Liver, and **B.** Spleen section, showing nuclei stained with DAPI, and background tissue fluorescence in the ZsGreen channel (ex 470 nm, em 510 nm).


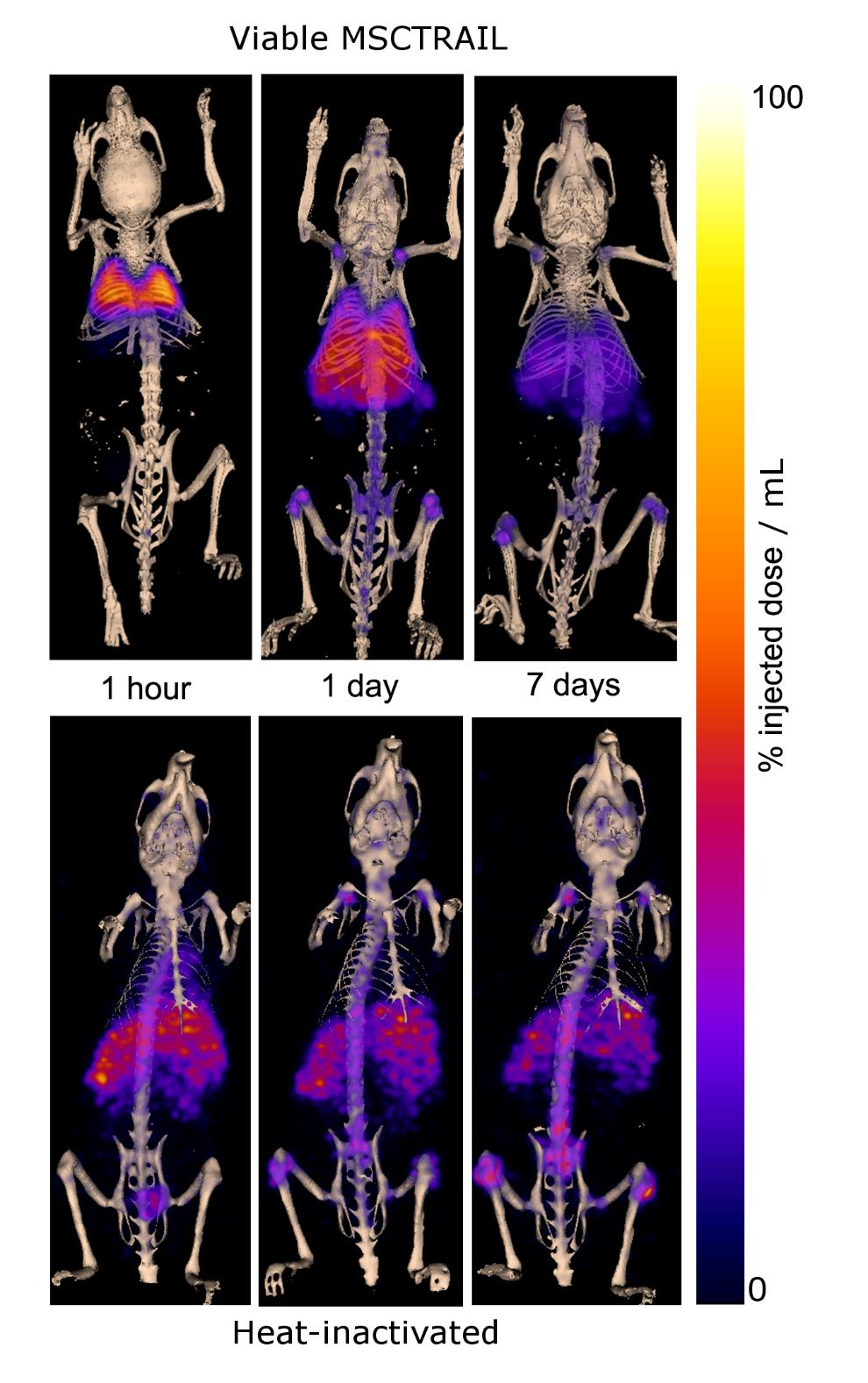


**Supplementary figure 14.** PET-CT maximum intensity projection images showing the distribution of signal at 1 hour, 1 day, and 7 days after intravenous injection of ^89^Zr-oxine labelled MSCTRAIL and ^89^Zr-oxine labelled cells that have been heat-inactivated (90^o^C for 20 minutes; 5 million cells in 500 uL PBS, 2 million injected).

**Table S1**. *Ex vivo* biodistribution data showing ^89^Zr activity measurements in the indicated organs at 10 days post injection of ^89^Zr-oxine labelled MSCTRAIL. Organs are ordered in descending values of % injected dose per gram tissue. N=3 animals and SD indicates standard deviation.

|  | % ID per gram | |  | % ID per organ | |
| --- | --- | --- | --- | --- | --- |
|  | Mean | SD |  | Mean | SD |
| Lungs | 78.18 | 26.31 |  | 12.36 | 4.32 |
| Spleen | 43.86 | 5.26 |  | 1.58 | 0.53 |
| Liver | 32.82 | 8.56 |  | 29.37 | 11.51 |
| Tibia | 8.35 | 1.93 |  | 0.94 | 0.19 |
| Kidneys | 4.31 | 0.69 |  | 1.24 | 0.27 |
| Tail | 0.99 | 0.54 |  | 0.45 | 0.27 |
| Heart | 0.74 | 0.29 |  | 0.09 | 0.03 |
| Thyroid and Salivary gland | 0.73 | 0.13 |  | 0.08 | 0.02 |
| Pancreas | 0.67 | 0.35 |  | 0.14 | 0.05 |
| Caecum | 0.36 | 0.06 |  | 0.09 | 0.02 |
| Large intestine | 0.31 | 0.07 |  | 0.16 | 0.08 |
| Stomach | 0.29 | 0.15 |  | 0.09 | 0.04 |
| Thigh Muscle | 0.29 | 0.11 |  | 0.05 | 0.03 |
| Small Intestine | 0.22 | 0.05 |  | 0.17 | 0.01 |
| Brain | 0.13 | 0.07 |  | 0.03 | 0.02 |

**Table S2**

Equivalent doses for a range of organs/tissues calculated using OLINDA in a male phantom model. The dose is expressed in in mSv per MBq injected, and also in mSv for 37 and 100 MBq injected activities.

| **Organ/Tissue** | **Equivalent Dose per MBq injected (mSv/MBq) for Adult Male** | **Equivalent Dose for 37 MBq injected activity (mSv) for Adult Male** | **Equivalent Dose for 100 MBq injected activity (mSv) for Adult Male** |
| --- | --- | --- | --- |
| Adrenals | 0.68 (0.56-0.87) | 25.0 | 67.6 |
| Brain | 0.07 (0.05-0.10) | 2.6 | 7.2 |
| Gallbladder Wall | 0.57 (0.46-0.67) | 21.2 | 57.4 |
| LLI Wall | 0.06 (0.04-0.07) | 2.1 | 5.6 |
| Small Intestine | 0.13 (0.10-0.16) | 4.8 | 13.1 |
| Stomach Wall | 0.35 (0.28-0.47) | 12.8 | 34.6 |
| ULI Wall | 0.17 (0.14-0.21) | 6.3 | 17.1 |
| Heart Wall | 0.80 (0.66-1.06) | 29.6 | 80.0 |
| Kidneys | 0.61 (0.48-0.73) | 22.6 | 61.0 |
| Liver | 1.86 (1.49-2.14 | 68.8 | 186.0 |
| Lungs | 5.09 (3.97-6.89) | 188.2 | 508.7 |
| Muscle | 0.23 (0.19-0.31) | 8.7 | 23.4 |
| Pancreas | 0.58 (0.46-0.76) | 21.3 | 57.5 |
| Red Marrow | 0.33 (0.27-0.44) | 12.1 | 32.7 |
| Osteogenic Cells | 0.55 (0.42-0.75 | 20.5 | 55.3 |
| Skin | 0.13 (0.11-0.18) | 4.9 | 13.3 |
| Spleen | 2.12 (1.49-3.36) | 78.3 | 211.7 |
| Testes | 0.02 (0.02-0.03) | 0.8 | 2.3 |
| Thymus | 0.51 (0.41-0.69) | 18.9 | 51.0 |
| Thyroid | 0.19 (0.15-0.25) | 6.9 | 18.6 |
| Urinary Bladder Wall | 0.04 (0.03-0.05) | 1.3 | 3.5 |

**Table S3.** Equivalent doses for a range of organs/tissues calculated using OLINDA in a female phantom model. The dose is expressed in in mSv per MBq injected, and also in mSv for 37 and 100 MBq injected activities.

| **Organ/Tissue** | **Equivalent Dose per MBq injected (mSv/MBq) for Adult Female** | **Equivalent Dose for 37 MBq injected activity (mSv) for Adult Female** | **Equivalent Dose for 100 MBq injected activity (mSv) for Adult Female** |
| --- | --- | --- | --- |
| Adrenals | 0.86 (0.70-1.11) | 31.8 | 85.9 |
| Brain | 0.08 (0.06-0.11) | 3.0 | 8.1 |
| Breasts | 0.53 (0.43-0.71) | 19.5 | 52.8 |
| Gallbladder Wall | 0.70 (0.56-0.82) | 25.8 | 69.6 |
| LLI Wall | 0.08 (0.06-0.10) | 2.8 | 7.6 |
| Small Intestine | 0.17 (0.13-0.21) | 6.2 | 16.8 |
| Stomach Wall | 0.46 (0.37-0.61) | 16.9 | 45.8 |
| ULI Wall | 0.21 (0.17-0.26) | 7.9 | 21.2 |
| Heart Wall | 1.05 (0.87-1.39) | 38.8 | 104.9 |
| Kidneys | 0.72 (0.56-0.86) | 26.5 | 71.7 |
| Liver | 2.39 (1.92-2.75) | 88.4 | 239.0 |
| Lungs | 6.58 (5.13-8.91) | 243.3 | 657.7 |
| Muscle | 0.30 (0.25-0.4) | 11.2 | 30.3 |
| Ovaries | 0.09 (0.07-0.12) | 3.3 | 9.0 |
| Pancreas | 0.76 (0.61-1.01) | 28.2 | 76.3 |
| Red Marrow | 0.38 (0.31-0.51) | 14.1 | 38.1 |
| Osteogenic Cells | 0.71 (0.54-0.97) | 26.3 | 71.2 |
| Skin | 0.16 (0.13-0.21) | 5.9 | 16.0 |
| Spleen | 2.57 (1.82-4.07) | 95.2 | 257.3 |
| Thymus | 0.61 (0.49-0.82) | 22.6 | 61.2 |
| Thyroid | 0.23 (0.19-0.32) | 8.6 | 23.4 |
| Urinary Bladder Wall | 0.05 (0.04-0.06) | 1.8 | 5.0 |
| Uterus | 0.08 (0.06-0.10) | 3.0 | 8.2 |

**Table S4.** Comparison of estimated human dosing between ^89^Zr-oxine cell injection and published studies using ^89^Zr-antibodies.

| **Source** | **Zr-89 Oxine TRAIL MSCs (37 MBq, Male)** | **Zr-89 Oxine TRAIL MSCs (100 MBq, Male)** | **Zr-89 Oxine TRAIL MSCs (37 MBq, Female)** | **Zr-89 Oxine TRAIL MSCs (100 MBq, Female)** | **Zr-89 Chimeric Monoclonal Antibody U36 (75 MBq, Female) [2]** | **Zr-89 Ibritumomab Tiuxetan (70 MBq) [3]** |
| --- | --- | --- | --- | --- | --- | --- |
| **Effective Dose per MBq injected (mSv/MBq)** | 0.87 (0.72-1.16) | | 1.12 (0.92-1.49) | | 0.66 | 0.55 |
| **Effective Dose (mSv)** | 32.2 | 87.1 | 41.4 | 111.8 | 49.5 | 38.5 |
| **Highest organ equivalent dose (mSv/MBq)** | 5.09 (3.97-6.89) Lungs | | 6.58 (5.13-8.91) Lungs | | 1.35 (Liver) | 1.36 (Liver) |
| **Highest organ equivalent dose (mSv)** | 188.2 | 508.7 | 243.3 | 657.7 | 101.3 | 95.2 |

Zr-89 Oxine MSC-TRAIL: Human Dosimetry Estimates based upon Pre-clinical Data

Pre-clinical data from the Centre for Advanced Biomedical Imaging (CABI) at UCL was used to estimate organ doses and an effective dose in adult humans (male and female) for the administration of ^89^Zr-Oxine labelled MSC-TRAIL.

The estimated effective dose is 0.87 mSv/MBq (range 0.72-1.16, n=3) for males, and 1.12 mSv/MBq (range 0.92-1.49, n=3) for females. For an administered activity of 37 MBq the effective dose is estimated as 32.2 (26.4-42.9) mSv for males, and 41.1 (34.0-55.1) mSv for females. For an administered activity of 100 MBq the effective dose is estimated as 87.1 (71.6-116.0) mSv for males, and 111.8 (91.9-149.0) mSv for females.

These are high doses in comparison with routine clinical PET imaging, for example an FDG PET is approximately 7.6 mSv for a 400 MBq administration [2]. The doses are more comparable with those reported for other Zr-89 labelled PET studies, but are still approximately 30-100% higher for equivalent administered activities [3] [4]. The highest organ equivalent dose is to the lungs, and is estimated to be 5.1 mSv/MBq (range 4.0-6.9) for males and 5.82 mSv/MBq (range 6.6-8.9) for females.

**Pre-clinical Data**

The pre-clinical data included the % of injected dose per gram of tissue (%ID/g) in mice for the Lungs, Liver, Spleen, Kidneys, and Bone, derived from PET/CT imaging at 1, 24, 48, and 168 hours post administration in three mice (for one mouse the 1 hour time point was not available). At 240 hours post administration the %ID/g was measured ex vivo for the following organs; Brain, Thyroid, Lungs, Heart, Liver, Spleen, Stomach, Small Intestine, Large Intestine, Caecum, Kidney, Muscle, Tibia, Pancreas, and Tail. Organ masses were also measured. The whole body weight of each mouse was estimated as 20 g.

For the PET/CT derived data, the decay corrected %ID/g for each organ (Lungs, Liver, Spleen, Kidneys, and Bone) was calculated for each mouse. The ex vivo data at 240 hours was not used for these organs, as the different method of measurement lead to a poor curve fit. The organs with PET/CT data accounted for 89-91% of the injected activity at 1 hour.

**Human Dosimetry Estimate**

The mouse data was extrapolated to human data using equations provided on p83 and 84 of Stabin, *Fundamentals of Nuclear Medicine Dosimetry* [5]. This included a conversion from mouse %ID/g to human %ID/organ using the measured mouse organ and whole-body masses and human phantom model masses from OLINDA, and a transformation of the timescale to account for differences in metabolic rate for species of different body mass [5]. The %ID/organ was normalised so that the total summed across the lungs, liver, kidneys, spleen, and bone was equal to 100%, as no rapid excretion is expected.

Curve fitting of time-activity plots was performed either in OLINDA (version 1.0, 2003) [6] or MATLAB, using either a multi-phase exponential decay model or a bi-exponential decay model that includes an uptake phase. The MIRD dosimetry methodology was applied to obtain the residence time for each organ.

The residence time was inputted into OLINDA. Organ doses were calculated using the adult male and adult female models. To match the OLINDA kinetics input form, all bone activity was assumed to be in the cortical bone.

1. Yuan Z, Kolluri KK, Sage EK, Gowers KH, Janes SM. Mesenchymal stromal cell delivery of full-length tumor necrosis factor-related apoptosis-inducing ligand is superior to soluble type for cancer therapy. Cytotherapy. 2015;17:885-96. doi:10.1016/j.jcyt.2015.03.603.

2. Comittee A. ARSAC Notes for Guidance Notes for Guidance on the Clinical Administration of Radiopharmaceuticals and Use of Sealed Radioactive Sources; 2016.

3. Borjesson PK, Jauw YW, Boellaard R, de Bree R, Comans EF, Roos JC, et al. Performance of immuno-positron emission tomography with zirconium-89-labeled chimeric monoclonal antibody U36 in the detection of lymph node metastases in head and neck cancer patients. Clinical cancer research : an official journal of the American Association for Cancer Research. 2006;12:2133-40. doi:10.1158/1078-0432.CCR-05-2137.

4. Rizvi SNF, Visser OJ, Vosjan M, van Lingen A, Hoekstra OS, Zijlstra JM, et al. Biodistribution, radiation dosimetry and scouting of (90)Y-ibritumomab tiuxetan therapy in patients with relapsed B-cell non-Hodgkin's lymphoma using (89)Zr-ibritumomab tiuxetan and PET. European journal of nuclear medicine and molecular imaging. 2012;39:512-20.

5. Stabin MG. Fundamentals of Nuclear Medicine Dosimetry: Springer; 2008.

6. Stabin MG, Sparks RB, Crowe E. OLINDA/EXM: the second-generation personal computer software for internal dose assessment in nuclear medicine. Journal of nuclear medicine : official publication, Society of Nuclear Medicine. 2005;46:1023-7.
